# Heatwave responses of Arctic phytoplankton communities are driven by combined impacts of warming and cooling

**DOI:** 10.1126/sciadv.adl5904

**Published:** 2024-05-17

**Authors:** Klara K. E. Wolf, Clara J. M. Hoppe, Linda Rehder, Elisa Schaum, Uwe John, Björn Rost

**Affiliations:** ^1^ Institute of Marine Ecosystem and Fishery Science, University of Hamburg, Hamburg, Germany.; ^2^ Environmental Genomics, University of Konstanz, Konstanz, Germany.; ^3^ Alfred Wegener Institute Helmholtz Centre for Polar and Marine Research, Bremerhaven, Germany.; ^4^ Helmholtz Institute for Functional Marine Biodiversity (HIFMB), Oldenburg, Germany.; ^5^ FB2, University of Bremen, Bremen, Germany.

## Abstract

Marine heatwaves are increasing in frequency and intensity as climate change progresses, especially in the highly productive Arctic regions. Although their effects on primary producers will largely determine the impacts on ecosystem services, mechanistic understanding on phytoplankton responses to these extreme events is still very limited. We experimentally exposed Arctic phytoplankton assemblages to stable warming, as well as to repeated heatwaves, and measured temporally resolved productivity, physiology, and composition. Our results show that even extreme stable warming increases productivity, while the response to heatwaves depends on the specific scenario applied and is not predictable from stable warming responses. This appears to be largely due to the underestimated impact of the cool phase following a heatwave, which can be at least as important as the warm phase for the overall response. We show that physiological and compositional adjustments to both warm and cool phases drive overall phytoplankton productivity and need to be considered mechanistically to predict overall ecosystem impacts.

## Teaser

Heatwave responses of Arctic phytoplankton are driven by warm and cool phases and differ from those to stable temperatures.

## INTRODUCTION

In a warming climate, extreme events such as marine heatwaves are becoming more frequent, intense, and longer-lasting (*1*). They often expose species to conditions beyond their tolerance thresholds with far-reaching consequences on entire ecosystems (*2*). Impacts of marine heatwaves include shifts in community composition, mass mortality of species, and severe biodiversity loss, up to the collapse of regional fisheries (*3*). In the Arctic, marine heatwaves have already increased in number over the past decades (*4*), and impacts are expected to increase further as temperatures rise and sea ice declines (*5*, *6*).

A broader understanding of the ecological impacts of marine heatwaves is still missing and so far limited to opportunistic observations of large-scale and long-term events (*7*) with a focus on higher trophic levels such as fish. For areas north of the Arctic circle, however, observations on marine heatwaves and their impacts hardly exist to date (*8*), and only very few recent ones have been described for Antarctica (*9*). To our knowledge, the northernmost warm water events for which direct biological impacts were recorded are in the Bering Sea, the Gulf of Alaska 2016 (*10*, *11*), and the North Atlantic 2012 (*12*), all of which reported northward shifts of species and changed migration patterns. The few described cases for changes at the lower trophic levels comprise shifts in phytoplankton species abundance and composition, including occurrences of toxic algal blooms (*13*, *14*). These changes propagated up the foodweb and led to shifts in important zooplankton species, eventually causing mass starvation or intoxication of higher trophic levels (*10*, *15*).

While there is little data on the responses of phytoplankton to heatwaves to date, general temperature effects on growth and other physiological processes are well described. Maximum growth rates are expected to generally increase with temperature (*16*, *17*), while warming beyond the optimum usually induces stress and decreases rates again (*18*). These temperature dependencies are typically derived from the responses of long-term acclimated laboratory strains, and it has been shown that they do not necessarily predict short-term responses under fluctuating temperature dynamics (*19*, *20*). Depending on the optimum ranges of an organism and time frames of exposure, variable temperature conditions can have negative impacts on phytoplankton productivity (*21*, *22*), e.g., because acclimation and selection act into different directions in warm and cool phases. Especially when exposed to extremely low or high temperatures, variability can also be beneficial and provide opportunities for physiological recovery or evolutionary rescue (*23*, *24*).

Interactions with other environmental and ecological factors in natural environments make variable temperature responses even harder to predict. Modeling and observations generally suggest that marine heatwaves cause a decline in primary productivity at lower latitudes, while productivity increases at higher latitudes (*10*, *25*, *26*). These opposing patterns are strongly connected not only to regional nutrient backgrounds but also to temperature-driven changes in nutrient and light availability due to increased stratification (*3*, *25*, *27*, *28*). Heatwaves at low latitudes often intensify nutrient limitation and decrease overall productivity, while heatwaves in comparably nutrient-rich high-latitude regions are expected to intensify blooms. This has indeed been observed in satellite-based chlorophyll *a* (Chl *a*) data in Antarctica (*29*), but high-resolution observations of the lower trophic levels during heatwaves are still lacking (*25*), especially for polar regions. The same is true for process understanding of phytoplankton responses to heatwaves, which is urgently needed to enable better predictions.

Experiments can provide answers to more targeted mechanistic questions regarding heatwave responses. The few existing studies on phytoplankton are yet difficult to compare because of very different experimental setups, ecological complexity, geographic regions, and measured parameters, and often do not specifically resolve the time period after a heatwave, i.e., when temperatures return to baseline conditions. Accordingly, they deliver very different results, ranging from detrimental [e.g., (*30*, *31*)] to beneficial effects on productivity or biomass production [e.g., (*32*)], and from strong stability to high sensitivity in taxonomic composition and diversity (*24*, *33*–*35*). This illustrates that responses to heatwaves are complex and need to be tested in view of their specific context, such as their seasonal and regional settings. One study has investigated polar phytoplankton under heatwaves by exposing isolated genotypes of an Antarctic diatom to heatwaves scenarios, finding increased mortality with increasing intensity and duration of heat exposure under nutrient limitation (*31*). Among the tested genotypes, however, they found large intraspecific variability, suggesting that, in an entire dynamic community, response variability is going to be even more relevant.

**Fig. 1.**
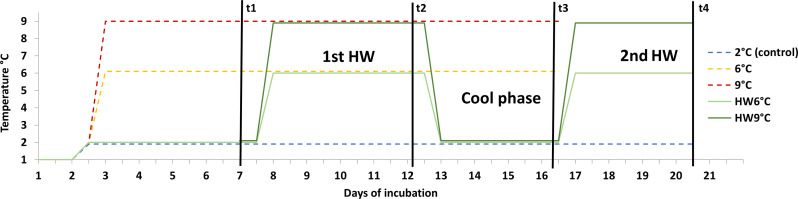
Experimental design. Overview of the temperature treatments (legend) and sampling and dilution points (t1 to t4) of the experiment. Three stable temperature treatments exposed phytoplankton communities to 6° and 9°C for 16 days and 2°C for 20 days. The two heatwave treatments (HW6°C and HW9°C) exposed communities to two consecutive 5-day heatwaves at 6° and 9°C, respectively, with a 3-day cool phase at 2°C in between and ran for a total of 20 days. Because of this design, the final time points are only directly compared between the heatwave treatments and stable 2°C at day 20 (t4) and between all stable treatments at day 16 (t3).

The aim of this study was to characterize the response of phytoplankton communities to elevated stable temperatures and to investigate whether such general temperature responses can help to anticipate responses to heatwaves of the same temperature range. We exposed triplicates of natural spring communities from coastal Svalbard (Norway) for 2 to 3 weeks to stable temperature treatments (2°, 6°, and 9°C), where 2°C acted as a control treatment, as well as to repeated 5-day heatwaves of differing intensities [HW6° and HW9°C; Fig. 1]. By excluding grazers and ensuring nutrient replete and stable light conditions, we focused on the effect of temperature only. To understand the dynamics and mechanisms during changing temperature regimes, we explicitly investigated the different phases of a heatwave toward their impact on the community. At several time points (t1 to t4), we measured an extensive set of parameters, including growth and productivity assays, stoichiometry, photophysiology, as well as species and intraspecific population composition. With this study, we deliver a rare record of the implications of heatwave scenarios for the most important traits of Arctic primary producers.

## RESULTS AND DISCUSSION

In the following, we will first present and discuss the responses to the stable temperature treatments in terms of their time-integrated outcomes, followed by the assessment of their temporal dynamics and their compositional development. Next, we will focus on the responses in the heatwave treatments and contrast those findings to the ones obtained from stable temperature treatments. Last, we will discuss what mechanistic understanding we have gained regarding heatwave responses and ecological implications arising from our findings. We would like to mention initially that, toward the end of the experiment, biofilm formation inside all incubation bottles could be observed (see details in Materials and Methods). While this potentially biased species composition toward benthic species, we are confident that it did not change overall outcomes or conditions of the incubations (e.g., light or nutrient regime).

### Even strongly elevated temperature stimulates community productivity

**Fig. 2.**
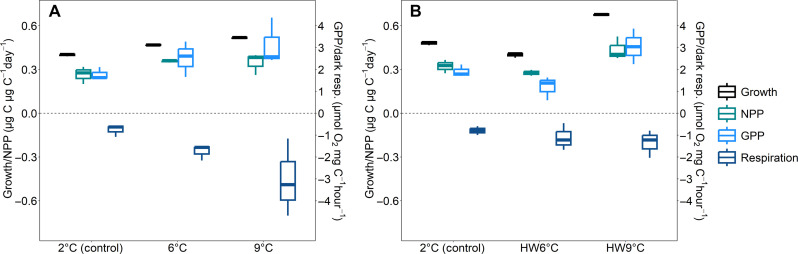
Weighted means over time of rate measurements. Growth (based on POC), NPP (based on ^14^C assays), GPP, and dark respiration (based on O_2_ evolution assays) as weighted means over the time of the experiment. (**A**) Stable temperature treatments up to day 16. (**B**) Heatwave treatments and stable 2°C up to day 20.

**Fig. 3.**
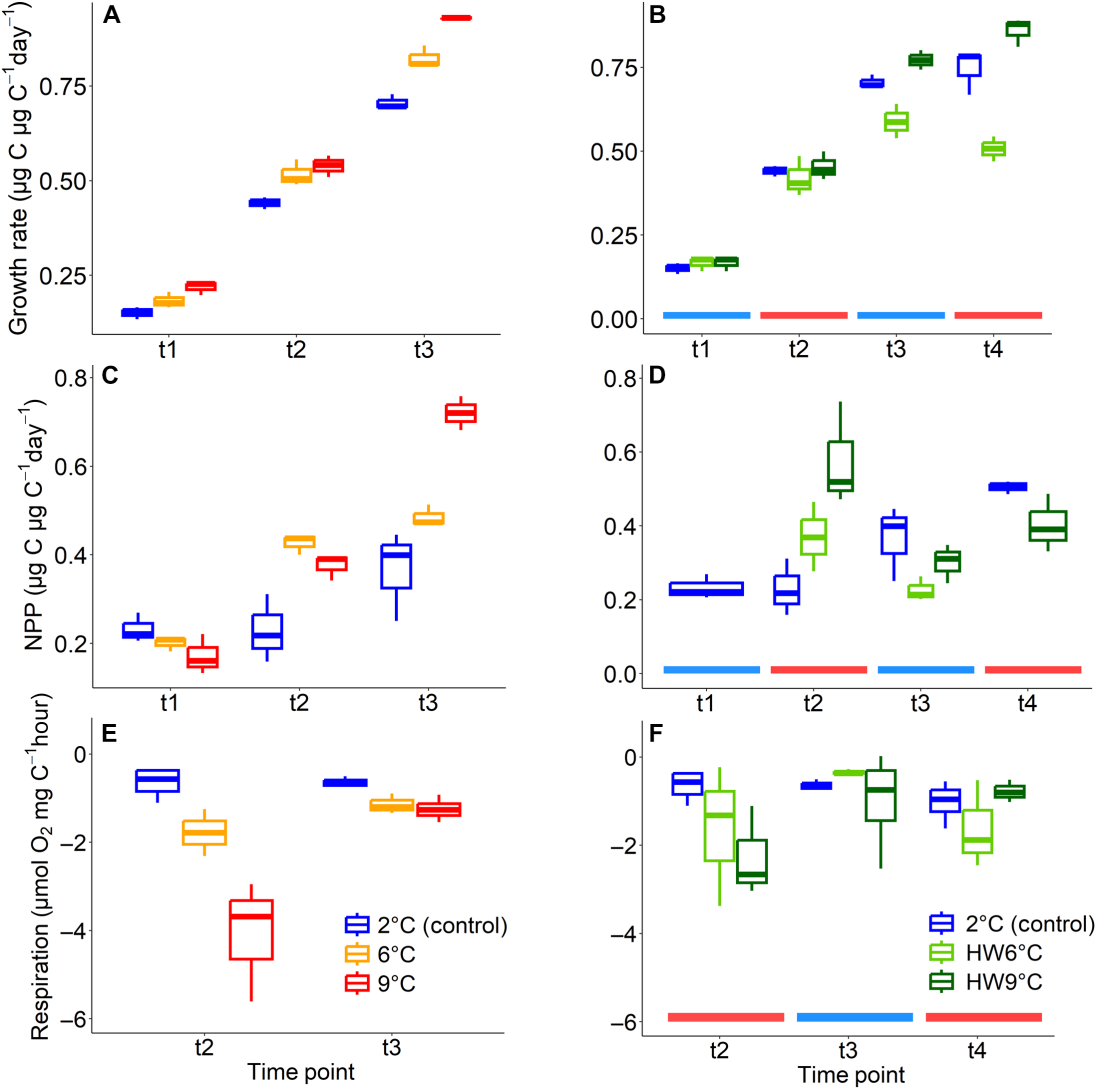
Time-resolved measurements. Growth rate (**A** and **B**), NPP (**C** and **D**), and dark respiration (**E** and **F**) at time resolution for the stable [(A), (C), and (E)] and the heatwave treatments [(B), (D), and (F)]. Blue and red lines at the bottom of the heatwave plots indicate where time points followed a cool or warm phase. NPP values for the heatwave treatments at t1 were adopted from the 2°C control treatment, NPP measurements of HW6°C at t4 were lost due to technical problems. Rates of O_2_ evolution were only measured from t2 onward because biomass was too low for accurate rate assessments before.

Growth rates increased with increasing temperatures under stable conditions. This was observed using weighted means to integrate over the entire experiment [linear mixed model (lme) for growth: *P* < 0.001; Fig. 2A and table S1] but also at time resolution (Fig. 3A, see below). The other two productivity measures, net primary productivity (NPP; ^14^C-based) and gross primary productivity (GPP; O_2_-based), also showed increasing trends with temperature (lme NPP: *P* = 0.02, GPP: *P* = 0.15; Fig. 2A and table S1). For growth and NPP, even the scale of increase with warmer temperatures was similar (6°C growth: +17%, NPP: +34% and 9°C growth: +30%, NPP: +31%). GPP increased at a higher mean rate (6°C: +41% and 9°C: +75%), but rates were also more variable (Fig. 2A). Although these three rate measurements have different units, and are based on different assumptions and potential biases by unaccounted processes (e.g., potential light and bacterial respiration in GPP), they consistently show similar positive trends with warming up to 9°C. While this is not unexpected in view of general knowledge on temperature effects on growth rates [e.g., (*16*)], it is impressive considering that we exposed communities to fairly extreme scenarios for high-latitude organisms and for a spring community at this specific location (see fig. S11). Growth rates yielded a Q10 value of 1.45 (±0.01) all the way up to 9°C. This value is close to Q10 estimates of the global average for phytoplankton (*36*), which include a large proportion of temperate species. This suggests that the sampled Svalbard community could indeed remain competitive compared to invading Atlantic species over the here tested temperature range. Our results also support the literature showing that many polar phytoplankton species live well below their optimum growth temperature (*37*–*39*).

The stimulatory effects of warming on productivity were observed despite even stronger increases in dark respiration relative to 2°C (6°C: +130%, 9°C: +300%, lme: *P* < 0.001; Fig. 2A and table S1). This is in line with studies showing that respiration is more sensitive to temperature change than photosynthesis in phototrophs [e.g., (*40*, *41*)] but is probably partly influenced by the fact that our community measurements comprise not only phytoplankton respiration but also heterotrophic organisms (here mainly bacteria). Nevertheless, we assume that primary producers were likely the dominant O_2_ consumers, which is corroborated by the largely congruent patterns of O_2_-based community GPP and ^14^C-based phototrophic NPP (Fig. 2).

The overall Chl *a*:POC ratio in the communities increased with temperature in the stable treatments (lme Chl *a*:POC: *P* = 0.002; fig. S1A and table S1), which is in line with a number of experimental studies (*30*, *42*–*44*). At the same time, however, electron transport rates at in situ light (isETR) showed a trend for lower values with increasing temperature (lme: *P* = 0.058; fig. S1C and table S1). Usually, the photosynthetic machinery is accelerated by warming, supported here by a trend of shorter reoxidation time at PSII (tau; fig. S1E), which enables higher productivity if sufficient light can be harvested. The decrease in electron transport at PSII (isETR) under warming may thus have been compensated by more reaction centers, reflected in the increase in Chl *a*:POC. This reorganization would make cells more flexible to opportunistically exploit higher light intensities when turnover rates in the Calvin cycle are accelerated by higher temperatures. Otherwise, responses in photophysiology were relatively minor, even during abrupt temperature ramps (fig. S7).

Time-resolved growth and productivity rates showed an increasing trend in all treatments over time (Fig. 3A). This is to be expected even in ambient treatments since the phytoplankton community adjusts increasingly to laboratory conditions, physiologically as well as by increasing contributions of fast-growing species under the replete nutrient and stable light conditions. In line with the time-integrated results (Fig. 2A), the temporal development of growth rates in the stable temperature treatments showed consistently larger increases at higher temperatures [linear model (lm) growth, 2°C slope = 0.055 day^−1^; 6°C slope = 0.063 day^−1^; 9°C slope = 0.07 day^−1^; table S2]. A similar development over time was visible in productivity measures (Fig. 3C and table S2), but not in respiration rates (Fig. 3E): In the 2°C treatment, dark respiration remained remarkably unchanged over the course of the experiment (lm on respiration without significant slope; table S2), apparently already being sufficiently acclimated to these temperatures before that time point. In the stable warm treatments, however, respiration rates were notably higher than those at 2°C early on [t2: 6°C: +173%, 9°C: +527%, analysis of variance (ANOVA): *P* = 0.003; table S2] and then decreased to values closer to 2°C at the last time point (t4: 6°C: +78%, 9°C: +94%, ANOVA: *P* = 0.02; table S2), suggesting a slow but substantial acclimation effect.

**Fig. 4.**
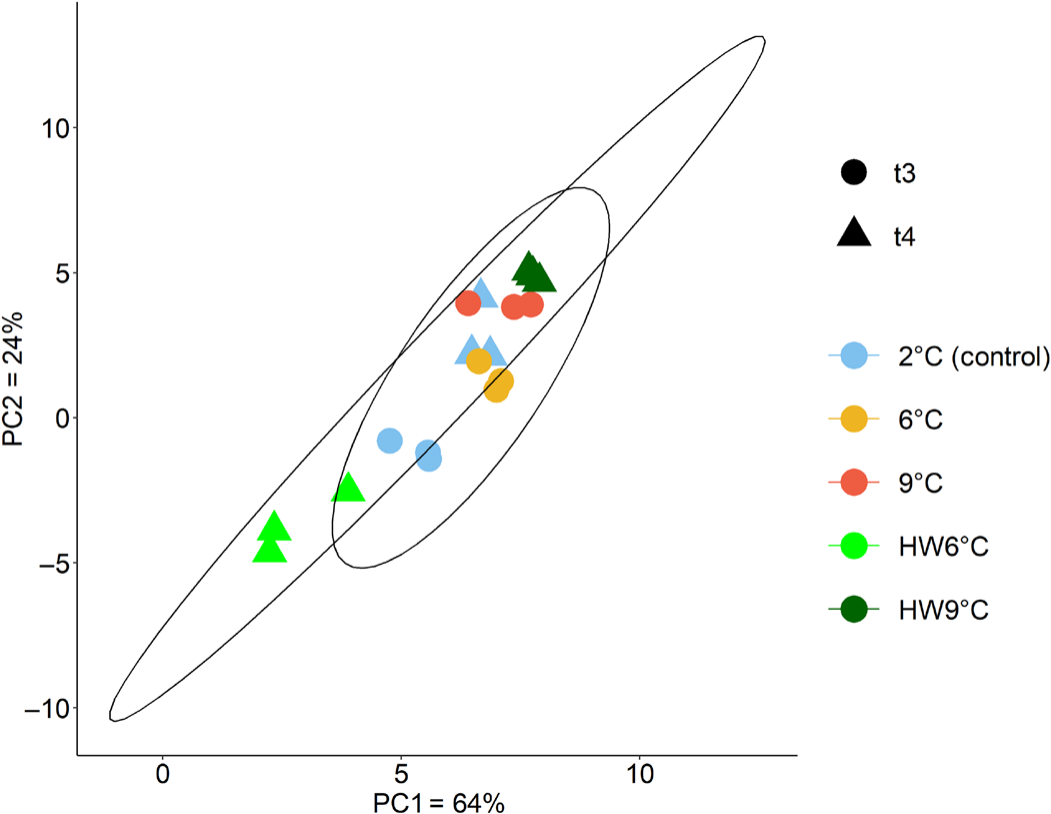
Species composition at the end of the experiment. Similarity of proportional species composition at the final time points of the experiment (stable treatments at t3 = 16 days and HWs at t4 = 20 days). The plot is based on a principal components (PCA) analysis of Aitchinson distances between 18*S* gene metabarcoding results of the three replicates of all treatments.

Next to physiological acclimation, sorting between species and strains can also contribute substantially to the overall response patterns (*45*). The experiment started off with a diverse pre–spring bloom community (figs. S2 and S3) dominated by diatoms, alongside dinoflagellates, haptophytes (*Phaeocystis*), and green algae (*Micromonas*). Note that community composition was assessed from 18*S* ribosomal RNA (rRNA) gene metabarcoding and is thus only meaningful concerning relative abundances within samples. By the end of the experiment, diatoms dominated all communities independently of the treatment [71 to 92% of taxonomically assigned amplicon sequence variants (ASVs)]. At the final time point, the species composition between the stable temperature treatments was distinct (PERMANOVA: *P* = 0.003; Fig. 4). Unexpectedly, dominant taxa were largely identical across treatments despite the large temperature differences, consisting mainly of the genera *Navicula*, *Fragilariopsis*, *Thalassiosira*, *Nitzschia*, and *Skeletonema* (fig. S3). The similarity between the treatments suggests that the majority of species retained similar competitive ranking to each other under stable temperature conditions between 2° and 9°C, suggesting a high resilience in species composition over the here tested temperature range. This resilience toward stable warming scenarios has been previously described for phytoplankton communities from different Arctic regions, which were furthermore concomitantly challenged with changes in carbonate chemistry and light levels (*46*).

Overall, the observed differences between our stable temperature treatments were much larger in bulk ecophysiological responses than in composition. We therefore conclude that changes in traits such as productivity were likely caused by physiological adjustments of the dominant members of the community in parallel, rather than by fundamental compositional changes. Our findings thus hint toward more general, cross-species physiological response patterns under stable warming.

### Heatwave responses are not predictable from stable temperature responses

In contrast to communities in the stable temperature treatments, responses to the heatwave treatments were much less linear and intuitive. Despite the stimulating effects at stable 6°C, the treatment with 6°C heatwaves (HW6°C) yielded overall lower productivity estimates than the stable 2°C treatment (growth: −17%, NPP: −14%, GPP: −37%; Fig. 2B). In the 9°C heatwave treatment (HW9°C), however, all productivity measures showed increased trends when integrated over time (growth: +43%, NPP: +35%, GPP: +59%; Fig. 2B) and even surpassed the rates at stable 9°C. Although unexpected, these trends are supported by three independent measurement techniques, depicting different biological aspects of productivity (biomass buildup, C fixation, and O_2_ production). The responses in respiration rates were more similar to the stable temperature exposures and showed increased trends in both heatwave treatments (Fig. 2B). Nonetheless, viewed in concert with the stable temperature treatments, the detrimental responses in the HW6°C treatment are especially puzzling. To understand the fundamental differences between the two heatwave treatments, it proved necessary to consider the different warm and cool phases separately over time.

In both heatwave treatments, it was not the warming itself that had the largest influence on biomass buildup. In fact, growth rates remained not only similar to those in stable 2°C during the initial phase but also even during the first heatwave (t2), i.e., in the first 5 days after temperature increased (Fig. 3B). Significant treatment differences in growth appeared only at the end of the cool phase (ANOVA growth t3: *P* = 0.002; table S2) and then continued to increase throughout the second heatwave (ANOVA t4: *P* < 0.001): While HW6°C communities showed substantially slower growth than 2°C control (second HW: −32%, Tukey’s test t4: *P* = 0.003; table S2), HW9°C communities grew faster (second HW: +15%, Tukey’s test t4: *P* = 0.07). In the NPP measurements, both heatwave communities showed increased rates at the end of the first heatwave already (ANOVA t2: *P* = 0.02; Fig. 3D), although this increase was significant only in HW9°C (+151%, Tukey’s test: *P* = 0.02; table S2). In the following cool phase (t3), net productivity of both heatwave communities tended to decrease to rates even below those observed at stable 2°C (t3 + t4, ANOVA not significant), with especially low rates in the HW6°C communities (Fig. 3D). Thus, in both productivity parameters, the heatwave communities showed the biggest divergence during the first cool phase (t3).

Respiration rates were only successfully recorded from day 11 onward (t2, after the first heatwave) but still show interesting temporal developments (Fig. 3F). During heatwave exposure, effects on respiration were similar in HW6°C and HW9°C and even to those in the stable treatments, although measurements yielded higher variability, causing differences not to be significant. During the first heatwave (t2), respiration increased in both heatwave treatments relative to 2°C (HW6°C: +152%, HW9°C: +248%). During the consecutive cool phase (t3), respiration fell back to levels at 2°C for both heatwave treatments and afterward, increased only in HW6°C during the second heatwave (t4, +58% compared to the 2°C control), but not in HW9°C (−24%). The trend of decreased respiration over time, which was observed in the stable warm treatments and in HW9°C, hints toward acclimation rather than stress accumulation through warm phases. Here, our data differ from findings on Antarctic diatom strains, where mortality was higher in cultures with repeated heat exposure (*31*).

Note that some of the differences in the dynamics of productivity might be caused by the fact that assay-based assessments (NPP, GPP, and respiration) display immediate responses of cells at a specific acclimation state, while growth rates represent an integrated signal of biomass produced over several days, which was formed by cells with potentially different physiological states during the process of acclimation. In HW6°C communities, the combination of lower NPP during the first heatwave (t2) and the cool phase (t3), along with the higher respiration during the second heatwave (t4), may thus have led to the overall decreased growth in this treatment, which started to be visible after the cool phase (t3). In HW9°C, on the other hand, the community had very high NPP rates during the first heatwave (t2), which may have carried over into the cool phase in the form of higher cell fitness or storage compounds. These “stored gains” (*24*) could have enabled increased growth even during the cool phase and, lastly, also an overall higher performance over the entire course of the experiment.

All treatments had distinct species compositions at the final time point, as described by their beta-diversities (PERMANOVA: *P* < 0.001; Fig. 4 and table S3). Their richness, however, as well as their dominant species were similar (figs. S2 and S3 and table S3). The notable exception was HW6°C, where the dominant genera differed from all other treatments (Fig. 4 and fig. S2): Here, *Navicula* was much less abundant than in any other treatment, while *Thalassiosira* and *Fragilariopsis* were more dominant. HW6°C was also the only treatment where a larger fraction of Chlorophyta (9% *Micromonas*) remained in the community. Because HW6°C communities were taxonomically distinct from all other treatments, and they were also unique in their lowered productivity and growth, it is likely that here compositional changes played a larger part in the overall response than for the other treatments. A closer look at the most dominant genera at the final time points reveals that some genera appeared to generally profit more or less from the applied treatments (fig. S4). Some profited from stable warming, such as species of *Fragilariopsis* and *Nitzschia*, while species of *Thalassiosira* and *Micromonas* declined under warming conditions. Fluctuating conditions as in the heatwaves, on the other hand, seemed to favor species of *Fragilariopsis*, as well as *Thalassiosira* in the intermediate heatwave, but not *Nitzschia*, indicating that also species dominance, here demonstrated by genera, can be poorly predicted from stable warming.

To also follow the intraspecific population composition, we used microsatellite PoolSeq barcoding [MPB; (*47*)] on one key diatom species, *Thalassiosira hyalina*, throughout the experiment. In addition to shifts between taxa, lineage sorting among diverse genotypes of the same species could be a powerful way of adaptation apart from physiological acclimation (*37*, *48*). On the basis of the allelic composition of both tested microsatellite loci, however, no directional shifts over time or among the treatments at the final time points were observed (fig. S6B). While the genus *Thalassiosira* decreased in abundance in all warmer treatments (fig. S6A), *T. hyalina* was likely even extinct at the final time point in the stable 9°C treatment as it was not detectable by MPB anymore. Overall, it does not appear that genotypic shifts within the species played a role in these communities.

Similar to the temporal development of growth rates, shifts in species composition of HW6°C also appeared during the cool phase (t3; fig. S5). Communities started to become distinctly different between treatments by the end of the first heatwave (t2, PERMANOVAs on beta-diversity t2 to t4 with *P* < 0.05; table S3). At this time point, communities in HW6°C were most similar to those at stable 2°C, while communities in HW9°C resembled those at stable 6°C (fig. S5). After the cool phase (t3), however, HW9°C communities approached the composition of stable 2°C again, while HW6°C shifted into a “new direction” and decreased in growth from then on. When comparing the two heatwave treatments, it is also notable that HW9°C communities remained more diverse after the first heatwave than HW6°C communities (t2; fig. S3), where some species relevant for buffering negative responses in the following cool phase may have been lost. Especially in fluctuating treatments, maintenance of higher diversity throughout a heatwave may provide a better basis for coping with the subsequent cool and warm phases.

### The interplay between warm and cool phases determines the overall heatwave response

The unexpected outcomes of the two heatwave treatments (Fig. 2) can only be explained by taking both, the warm and cool phases into account, as well as their interplay. Ecologically motivated heatwave experiments often report carryover effects and low recovery of phytoplankton composition after a warm phase [e.g., (*32*, *49*)], but more mechanistic studies classically focus on the high-temperature phase [e.g., (*30*, *50*)]. Little attention has been paid to physiological responses during the phase when temperatures return to more ambient levels [but see (*31*)], although this may interrupt an ongoing acclimation process and respectively start a new one in an opposite direction. Our overall data on ecophysiological and taxonomic changes suggest that a cool phase following a heatwave can be at least as decisive a driver as the warm phase itself. A cool phase can even act as an additional stressor rather than a relief to organisms and change compositional trajectories as argued above for the case of HW6°C. Similar seemingly “counterintuitive” responses were also described on a physiological level for phytoplankton by Rehder *et al.* (*43*), who found abrupt temperature decreases to cause transient imbalances in subcellular processes that can be even larger than those caused by warming. Temperature optima of polar species are often found above ambient temperatures (*37*, *39*), which may contribute to this effect, but the physiological impacts of cooling have to our knowledge rarely been investigated.

While compositional changes played an unexpectedly minor role in the majority of our treatments, it likely had a considerable impact on the responses of HW6°C, especially from the cool phase on. This example illustrates how small selective shifts at one time point may translate into large effects on productivity and composition later on, an aspect especially hard to foresee for exponentially growing organisms under fluctuating conditions. During the different temperature phases of a heatwave scenario, natural communities are likely confronted with opposing acclimation and selection pressures, which can shift or reduce the diversity during one phase in a way that is decisive for the next. While most dominant taxa in our study appear to have similarly large plastic ranges in their stable temperature response, fluctuations can apparently change this picture, unless heatwave-like fluctuations become the “new normal” and species’ abilities to withstand fluctuations are what is being selected for in a future ocean.

### Ecological implications of heatwave responses may be unexpectedly large and complex

The implications of physiological rate changes in exponentially proliferating organisms are often not easy to grasp but are ultimately the drivers of bloom dynamics and biogeochemical cycling (*51*–*53*). For better illustration, we calculated theoretical biomass accumulation based on growth rates for each of our treatments without dilution (i.e., as in a bloom modeled without loss terms; fig. S8). After 14 days of stable warm temperatures, biomass production would thus be stimulated by +280% at 6°C and +560% at 9°C compared to 2°C. After 20 days with two 5-day heatwaves (i.e., 10 days warm and 10 days cold), communities would have experienced a smaller increase under 9°C heatwaves compared to stable 2°C (+60%) and a large decrease under heatwaves of 6°C (−86%). Of course, this upscaling exercise applies vast simplifications, e.g., it does neither account for potential resource limitation nor loss terms such as sinking or grazing. Although effects of heatwaves on grazing are still not well understood (*54*) and likely depend on many concomitant factors, first evidence from longer heatwaves hints toward an increase in zooplankton abundance through reduced top-down control by forage fish during warm phases (*55*, *56*). Our data nevertheless demonstrate the large potential ecological implications of bottom-up changes in biomass buildup of the observed scale over a bloom situation, underlining the need for a better understanding of these short-term temperature events. Especially if compositional changes between taxonomic groups appear, as in HW6°C with a larger relative abundance of Chlorophyta compared to diatoms, impacts on biogeochemistry and trophic transfer efficiency along the food chain can be expected (*57*). Whether these taxonomic shifts are a cause or an effect of the observed physiological responses cannot not be distinguished from this study. Further physiological studies on single species under heatwaves might help to shed light on this question, but here, compositional shifts were apparently driven more by the fluctuation of temperature than its absolute value. This in mind, note that responses to fluctuating temperature are not independent of the thermal history and the optimal temperatures of different species, which is why elevated future mean temperatures likely also have an effect on heatwave responses (*58*).

The overall increased Chl *a*:POC ratio at higher temperatures in photosynthetically dominated communities could also have important implications. A systematic influence of temperature on Chl *a*:POC ratios, as also suggested by physiological theory (*59*), could have large implications for modeled biomass predictions because observations of altered primary production are often based on Chl *a* as proxy for phytoplankton biomass. Effects of elevated temperature on phytoplankton biomass in the field [e.g., (*29*)] could thus be overestimated. Furthermore, NPP measurements, which are usually normalized to Chl *a*, would be inconsistent with C-based estimates at different temperatures. An illustration of this effect can be found in fig. S9, where NPP results of the present study are shown normalized to C and Chl *a*, with very different trends due to the shifts in underlying Chl *a*:POC ratios under different treatments.

Lastly, please note that this study was designed to single out and understand the effects of temperature alone on Arctic phytoplankton communities. To achieve a complete assessment of heatwave and temperature effects in a realistic context, interactions with other bottom-up factors such as nutrients and light but also top-down impacts such as grazing should be included. Nutrient regimes are likely very crucial for heatwave responses of phytoplankton, especially as they are expected to change alongside with warming (*2*) and can drive species composition and response sensitivity in systematic ways (*3*, *60*). More specifically, heatwaves themselves can impede nutrient supply through stratification (*25*), and nutrient limitation was found to lower the temperature optimum for phytoplankton growth, making them more prone to heat stress (*24*, *60*, *61*). In the Arctic, temperature-nutrient interactions are thus likely most relevant during summer when heatwaves and nutrient-limitation coincide. Such mechanistic understanding will be necessary to identify the most important drivers and to disentangle the complex interactions between them.

In conclusion, we found that stable warming strongly increased growth and productivity in spring phytoplankton communities from Svalbard, although the temperatures were far above organisms’ thermal history at this location (see fig. S11). Responses to short-term heatwaves proved less intuitive as they were driven by a complex interplay of warming and cooling effects, and shifts in community composition and physiology were carried over from one temperature phase to the next. Notably, response differences and shifts in composition became especially apparent during cool phases and were not necessarily larger at more extreme heatwave temperatures. Our study shows that temperature increases can cause positive or negative effects on productivity, depending on whether exposure takes place uniformly or oscillates as a heatwave and includes cool phases. Therefore, our knowledge on responses under stable warming cannot simply be transferred to anticipate effects of heatwave scenarios, which consist of warming and cooling. Better predictions on heatwave responses require improved mechanistic understanding and will depend on the properties of temperature fluctuation itself (intensity and reoccurrence, as well as duration of the temperature exposure). The cool phase after a heatwave appears to have a decisive impact, which is at least as big as the effect of the warm phase, and should receive more scientific attention in the future.

## MATERIALS AND METHODS

### Initial sampling and culturing conditions

Initial phytoplankton assemblages were sampled in April 2021 from the Kongsfjord, Svalbard, Norway (mid-fjord station KB3, 78°55′N, 11°56′E), in a pre–spring bloom setting. Seawater temperature was −0.1°C, Chl *a* concentration was 0.15 μg liter^−1^, while nitrate, phosphate, and silicate concentrations were 10.5, 0.7, and 5.0 μM, respectively. Seawater was pumped up from a depth of 19 m using a monsoon pump (Mega-Thyphoon, Proactive Environmental Products; flow rate, approximately 4 liters min^−1^) and directly filled into 4-liter polycarbonate incubation bottles (Nalgene) after passing through a 100-μm nylon mesh to remove large grazers from the community. To ensure homogenous filling while drifting with the boat, all culture bottles were filled halfway first before starting to fill them completely. From the same location, depth, and day, 300 liters of seawater was pumped up, prefiltered (0.2 μm), and stored in the dark and cold (2°C) for dilutions throughout the experiment.

The incubation bottles were then directly transferred to a temperature-controlled room at the Kings Bay AS Marine Laboratory, Svalbard, where they were exposed to constant 24-hour daylight of 31 ± 0.8 photons m^−2^ s^−1^ using a full-spectrum white Mobile LED Illumination System (CLF PlantClimatics GmbH). Light conditions were chosen in accordance with average in situ conditions at that time of the year (last sunset in mid-April) at a depth of 10 to 20 m (according to own measurements on site). For additional temperature control, bottles were placed inside five Plexiglas aquaria, in which water temperatures were adjusted using immersion thermostats (JULABO GmbH) and monitored throughout the experiment using a temperature logger (Almemo 2890, Ahlborn). The incubation bottles were continuously sparged (approximate flow rate of 100 ml/min) with ambient air delivered through sterile 0.2-μm air filters to keep cells in suspension and carbonate chemistry stable.

To ensure exponential growth of the phytoplankton communities throughout the experiment and to avoid nutrient limitation owing to high cell densities, cultures were diluted to ~2 μg Chl *a* liter^−1^ (mean 1.8 ± 0.04 μg liter^−1^) after each sampling time point, i.e., at three to four time points throughout the experiment (Fig. 1, t1 to t4, and fig. SI12). After each dilution, nutrients were added to reach values slightly above natural concentrations (averages: 17 μM NO_3_, 3 μM PO_4_, and 15 μM SiO_2_). At the initial time point, NO_3_ was accidentally added in excess in all treatments, reaching 65 to 85 μM. Throughout the experiment, nutrients were sampled approximately every 2 days, directly measured using a QuAAtro39 analyzer (Seal Analytical Limited) on site, and refilled when running low. Toward the end of the experiment, there were a few time points when nutrients reached potentially limiting conditions (<8 μM NO_3_) in the fasted growing treatments (e.g., t3 in stable 9°C and t4 in HW9°C), periods that were yet always short in time, at the very end of the experiment (for more details, see fig. S13) and not in all biological replicates, which showed nevertheless very similar responses. We therefore do not see evidence for nutrient conditions affecting the communities in a crucial way.

### Experimental design and time frames

Directly after the initial sampling, all 15 culture bottles were incubated in the laboratory at 1.5°C for an initial adjustment phase for 2.5 days (Fig. 1), after which they were transferred in random triplicates to one of the five different temperature treatments: stable 2°C, acting as a control treatment (1.96° ± 0.3°C), stable 6°C (5.8° ± 0.2°C), stable 9°C (8.81° ± 0.1°C), and two heatwave treatments, each with two consecutive 5-day heatwaves, with a 3.5-day cool phase in between (2.0° ± 0.2°C; Fig. 1). In treatment HW6°C, both heatwaves were set to 6°C (5.94° ± 0.03°C), and in treatment HW9°C, heatwaves were set to 9°C (8.87° ± 0.3°C). For the initial 5 days at 2°C (up to dilution time point 1), there were only three heatwave replicates, which were then split up after the first dilution into six bottles for HW6°C and HW9°C. All temperature transitions were reached by a 1°C per hour temperature ramp by either increased heating of the thermostats or addition of ice to the aquaria under continuous mixing.

Throughout the course of the experiment, the incubation bottles accumulated biofilms on their inside surfaces (benthic diatoms, dominated by *Navicula* sp.). The extent of biofilm formation was approximately congruent with the growth rates in the different treatments, with stable 9°C and HW9°C having the strongest biofilm, followed by stable 6°C, and HW6°C and the 2°C control treatment with the weakest biofilm. Because the cultures were diluted regularly, and the surface growth was strongest on the bottom side of the bottles, the biofilms never reached an extent interfering with the experimental conditions (e.g., light reduction). At the last dilution time point for each treatment (day 16 for 6° and 9°C and day 20 for the others), we gently removed those biofilms with a magnetic stirrer from the bottle surfaces, followed by rinsing the bottle with hot water, distilled water, and cooled growth medium before aliquots of the treatment were lastly reinoculated into temperature-adjusted medium again. Despite our efforts, all cultures showed strongly decreased performance after this procedure (see fig. S10), which is why we decided to remove subsequent data from our analysis, leaving a total duration of 16 days for 6° and 9°C (t3) and 20 days (t4) for the other treatments. The final time point for the heatwave treatments was thus the end of the second heatwave, not comprising the final cooling phase. Next to monitoring of nutrients and Chl *a* content every other day, sampling for a range of parameters took place just before each dilution of the culture bottles by gentle filtration (<200 mbar) of water samples in a 5°C room at low light or by conducting dedicated assays (see below).

### Growth and elemental composition

Specific growth rates μ (day^−1^) between sampling/dilution time points were calculated on the basis of measured Chl *a* and particulate organic carbon (POC) concentrations at the dilution time points (t1 to t4; Fig. 1). We applied an exponential growth function to the respective values with μ = ln(*Nt*) − ln(*N0*)/Δ*t*, where *Nt* is the value at the time of sampling (e.g., t4), *N0* is the value after the last dilution (e.g., t3), and Δ*t* is the time passed in days between those time points. *N0* was calculated from the measured value just before dilution (e.g., t2), and the exact volume the bottle was diluted by in milliliters.

For determination of total Chl *a*, 100 to 200 ml of water sample were filtered gently (<200 mbar) and under cold conditions onto precombusted (15 hours, 500°C) glass-fiber filters (GF/F, Whatman, United Kingdom). Chl *a* samples were immersed in cold 90% acetone and shredded with glass beads (0.5 to 1 mm in diameter) in a homogenizer (Precellys Evolution, Bertin Technologies, France), before being extracted overnight at −20°C. Chl *a* concentrations were measured fluorometrically (Trilogy, Turner Designs, United States), including an acidification step (1 M HCl) to determine phaeopigments (*62*).

Samples for POC and particulate organic nitrogen were taken in the same way as those for Chl *a*. Filters were directly frozen at −20°C and later acidified and dried overnight at 60°C before elemental analysis was performed using a gas chromatograph CHNS-O elemental analyzer (Euro EA 3-,000, HEKAtech). All raw values were corrected by the mean of blank filters (*n* = 6) handled alongside the sampling process.

### Species and population composition

Species composition was assessed by 18*S* rRNA metabarcoding. In the same filtration setup as described above, 400 to 500 ml of samples was filtered on 0.8-μm PC filters (Nucleopore, Whatman, United Kingdom), and immersed in 650 μl of preheated extraction buffer [SL1 of the NucleoSpin Soil extraction kit (see below) at 50°C] to be stored at −20°C until further analysis. After thawing and cell disruption with a MagNa Lyser (Roche Diagnostics, Switzerland), DNA extraction was performed according to the manufacturer’s protocol using the NucleoSpin Soil extraction kit (Macherey-Nagel GmbH, Germany). Amplicon libraries of the V4 region (18*S* rRNA gene) were generated using the standard 16S Metagenomic Sequencing Library Preparation protocol (16S Metagenomic Sequencing Library Preparation, part no. 15044223 Rev. B. Illumina, United States) using the forward primer CCAGCASCYGCGGTAATTCC and reverse primer ACTTTCGTTCTTGAT (*63*). Single samples were indexed using the Nextera XT Index Kit v2 primers (Illumina, United States) and pooled for sequencing on a MiSeq sequencer (Illumina, United States). Results were demultiplexed, and FASTQ sequence files were generated using the Generate FASTQ workflow of the MiSeq sequencer software, yielding a total of ~10 × 10^6^ raw amplicons. Primers were removed with cutadapt v2.8 (*64*), and further processing of the sequence data was performed using the DADA2 R package v1.18 (*65*). Reads were trimmed [forward reads after 240 to 260 base pairs (bp) and reverse reads after 200 to 210 bp] and denoised before paired-end reads were merged (minimum overlap 50 bp and no mismatches), and predicted chimeras were removed, yielding a total of ~6.8 × 10^6^ filtered amplicons (table S4). Taxonomic assignment of the resulting ASVs was performed using the reference databases PR2 (v4.12.0). For downstream analyses in the software R (version 4.3), nonphototrophic taxa were removed, as well as ASVs with a count of less than 10 reads in replicate sample means. Sequencing depth was checked using rarefaction curves, and raw data were normalized using a scaling with ranked subsampling procedure (srs) for further analysis.

Intraspecific population composition of the diatom *T. hyalina* was assessed via MPB, following the protocol as described by Wolf *et al.* (*47*). The sample (500 to 700 ml) was filtered on 10-μm PC filters (Nucleopore, Whatman, United Kingdom) and immersed in 650 μl of warm extraction buffer to be stored at −20°C until further analysis. DNA was extracted and applied in triplicate in a first-stage amplicon polymerase chain reaction (PCR) with the microsatellite primers ThKF3 and ThKF7. PCR products were visualized on an agarose gel, and bands at the approximate size of the microsatellite sequences were manually excised and purified (PCR Clean-up Kit, Macherey-Nagel, Germany). Single samples were indexed using the Nextera XT Index Kit v2 primers (Illumina, United States) and pooled for sequencing on a MiSeq sequencer (Illumina, United States). Demultiplexing and FASTQ sequence generation were performed using MiSeq Reporter software, yielding a total of 7 × 10^6^ raw amplicons for ThKF3 and 10 × 10^6^ for ThKF7. Amplicon contingency tables were constructed for each primer set using an in-house modified metabarcoding analysis pipeline, including cropping, trimming, merging, and truncating amplicons, as well as several feature filters for quality control (filtered amplicons ThKF3: 2.4 × 10^6^ and ThKF7: 3 × 10^6^). Resulting amplicon contingency tables were then further filtered for correct microsatellite sequences and minimum abundance, and amplicon numbers were standardized. The analysis of the processed data was performed using principal components analyses (PCAs) of all analyzed samples, as well as sample distance matrices.

### Physiological assays

Photophysiological parameters were measured via variable Chl *a* fluorescence of photosystem II (*66*) using a fast repetition rate fluorometer (FRRf, FastOcean PTX; Chelsea Technologies, United Kingdom) in combination with a FastAct Laboratory system (Chelsea Technologies). Temperatures inside the measurement chamber were adjusted by continuously pumping water of the respective aquarium into the FastAct chamber around the cuvette. Samples were dark-acclimated for >15 min before each measurement. Because it was not possible to run full photosynthesis-irradiance (PI) curves for all replicates on a single day, we measured samples of each bottle in a reduced PI protocol, collecting repeated measurements after 10 min of exposure to no light, experimental light (~30 μmol m^−2^ s^−1^), and oversaturating light levels (~600 μmol m^−2^ s^−1^) to inflict a light stress response. This allowed us to retrieve a basic set of parameters for all replicates, including Fv/Fm, connectivity of photosystems, and reoxidation time at PSII (tau) from dark-acclimated samples as well as isETR at experimental light levels (*67*, *68*). In addition, we recorded immediate photophysiological responses in the HW9°C treatment during the phases of warming and cooling of both heatwaves. When temperatures were increased/decreased at 1°C hour^−1^, at each step, a fresh sample was dark-acclimated and measured ~15 min after the temperature change with the reduced PI protocol as above. This yielded four “temperature ramps” from 2° to 9°C and the reverse.

NPP was measured in duplicate by 24-hour incubation with a NaH^14^CO_3_ spike (53.1 mCi mmol^−1^ or 2.109 megabecquerel mol^−1^ stock; PerkinElmer) under the respective treatment conditions. We used the same protocol as in Hoppe *et al.* (*46*). In short, 20-ml aliquots were incubated after addition of 10 μCi NaH^14^CO_3_ (specific activity of 0.5 μCi ml^−1^). Total amounts of added NaH^14^CO_3_ (DPM_100%_) and blank values (DPM_0%_) were determined through 0.5-ml aliquots that were immediately added to 1 M NaOH and 6 M HCl, respectively. DPM_100%_ samples were measured after 2 hours, and DPM_0%_ samples were handled alongside the experimental samples. After 24 hours, incubated samples were filtered onto GF/F filters, acidified with 0.5 ml of 1 M HCl, and left to degas overnight. Ten milliliters of scintillation cocktail were added (Ultima Gold AB, PerkinElmer), and samples were vortexed and left to stand in the dark for approximately 12 hours before counting on the liquid scintillation counter (DPM_sample_) using an automatic quench correction and a counting time of 5 min. NPP [μg C (μg Chl *a*)^−1^ day^−1^] was calculated as *NPP* = ([*DIC*] × (*DPM*_sample_ − *DPM*_0%_) × 1.05)/(*DPM*_100%_ × *t* × [*Chl* *a*]), where [*DIC*] and [*Chl* *a*] denote the concentrations of dissolved inorganic carbon and Chl *a* in the sample, respectively. DPM_sample_ denotes the disintegrations per minute (DPM) in the samples, DPM_0%_ and DPM_100%_ are the DPM of the blank and total amount of NaH^14^CO_3_, and *t* is the duration of the incubation. The value of 1.05 was used to correct for fractionation against ^14^C relative to ^12^C (*69*). Since, in all treatments, the Chl *a*:POC ratio increased during the first 10 days of the experiment (fig. S1A), likely due to acclimation to the stable but low light intensities in the experimental setup, we normalized NPP to POC instead of Chl *a*. Chl *a*–specific rates were converted to C-specific rates by using the measured Chl *a*:POC ratio for each replicate bottle at the respective time point.

Measurements of O_2_ evolution were performed in discrete assays in 20-ml gas-tight glass vials equipped with oxygen and temperature sensor spots, in combination with a Firesting-PRO station (PyroScience, Germany). Two samples of each biological replicate were incubated headspace-free for 24 hours under experimental conditions inside the water tanks (alongside the respective ^14^C-based assay vials), one in the light and one in darkness. The O_2_ concentrations in each vial were measured at a start and end time point for >10 min until the signal was stable while gently mixing the sample using a small magnetic stirrer. One additional replicate per measurement day was incubated under constant O_2_ logging as a technical control. All sensors were two-point calibrated at 0 and 100% atmospheric O_2_ for each temperature. O_2_ calibrations (0%) and temperature calibrations were performed before the start of the experiment by adding sodium sulfate to distilled water until saturation. Calibrations (100%) were performed once before and once during the experiment (t3) for each temperature in seawater. Temperature-corrected O_2_ values were read out at a mean of 2 min after the measurement stabilized at the beginning and endpoint. The difference of these two values were then divided by the exact incubation time to derive rates of net O_2_ evolution per hour, which was then normalized to the initial Chl *a* concentration of the sample. GPP was calculated by adding the rate of respiration measured in the dark to the net O_2_ evolution measured in light. Outlier values were identified using Dixon’s test (*P* < 0.1) and excluded from further analysis (5 of 94 measurements). Measurements at time point 1 were discarded for all treatments because biomass was too low to produce meaningful signals. Because of the variability in Chl *a*:POC ratios throughout the experiment, we transformed all productivity measurements to rates per POC rather than Chl *a* using the ratio of the closest time point where both parameters were measured.

Because assays were time-consuming and some instrumentation hardware was limited (O_2_ optode setups and FRRf), some of the assays had to be performed a day before or after the dilution time points (t1 for 6° and 9°C: 1 day before and t2 to t4 2°C: 1 day after). In those cases, all assay measurements were started on the same day, alongside which additional Chl *a* samples were analyzed, and Chl *a*:POC ratios of the respective time point were applied for biomass normalization.

### Statistical analysis

Since heatwave and stable warm treatments were running for different time frames (16 and 20 days), only their trends and not their absolute values can be directly compared. Therefore, they are depicted in separate graphs along with the 2°C control treatment after the respective time. To assess treatment effects across the entire experiment, we estimated the cumulative responses as weighted means of all time points for all parameters, thus integrating the overall effect throughout the experiment rather than only taking the final measurements into account. We did this by calculating a weighted mean of all time points according to the number of days passed between them. For statistical testing, we used linear mixed-effect models (lme4 R package version 1.1.3) on data from all time points, with treatment as fixed effect and time and replicate as random effects. A null model was run on the same data without treatment as fixed effect. The treatment was reported as having a significant effect if the comparison of the two models (ANOVA) yielded a significant result, and χ^2^, degrees of freedom, and *P* value were reported. Post hoc tests were performed using pairwise comparisons of estimated marginal means (emmeans package, version 1.8.7), and Bonferroni correction was applied to control for multiple testing. Model assumptions of linearity and homoscedasticity were verified for each dataset. To meet these assumptions, respiration rate data and growth rates of the heatwave treatments were log-transformed before analysis.

For the time-resolved data, we used one-way ANOVAs to identify differences between treatments at specific time points in question or between time points within a treatment (e.g., in response to temperature change). For a further analysis of the separate treatments, we used Tukey’s post hoc tests. Furthermore, we used linear models (lm) for regression analysis over several time points. Also here, model assumptions of linearity and homoscedasticity were verified in each case.

To analyze species composition data, srs-normalized asv data were used for composition plots showing the top five genera (fig. S2) and to calculate species richness and Shannon index as measures of alpha-diversity (fig. S3). Note that community composition was based purely on 18*S* rRNA gene metabarcoding, which is not a strictly quantitative measure and can be subject to quantitative distortions. Since all treatments contained largely the same genera at the respective time points, however, results contain meaningful information on relative abundance dynamics. Following Gloor *et al.* (*70*), beta-diversity was estimated through pairwise dissimilarity matrices using Aitchison distances, i.e., the Euclidean distance of centered log-ratio–transformed raw data and was visualized through PCA. Treatment differences were tested by PERMANOVA analysis.

To assess the effects of different treatments in a bloom setting, responses were also compared as upscaled biomass buildup of the initial community (fig. S8). This theoretical biomass buildup was calculated on the basis of the POC content of the phytoplankton community at t0 and then modeled for each replicate using an exponential growth function and the respective growth rate over the days of incubation from one time point to the next. This result was then used as the base for the next time point and, therefore, yielded exponential accumulation until the end of the experiment.

## Supplementary Materials

### This PDF file includes:

Figs. S1 to S13 Tables S1 to S4 References
