# Supplementary material for: Heatwave responses of Arctic phytoplankton communities are driven by combined impacts of warming and cooling
Source: Sci Adv. 2024 May 17;10(20):eadl5904. doi: 10.1126/sciadv.adl5904 (PMC11100554; doi:10.1126/sciadv.adl5904)
Supplement: Supplementary file 1 — Figs. S1 to S13 Tables S1 to S4 References [file sciadv.adl5904_sm.pdf]

Science Advances
AAAS

Supplementary Materials for

Heatwave responses of Arctic phytoplankton communities are driven by combined impacts of warming and cooling

Klara K. E. Wolf *et al.*

Corresponding author: Klara K. E. Wolf, klara.wolf@uni-konstanz.de

Sci. Adv. **10**, eadl5904 (2024)
DOI: 10.1126/sciadv.adl5904

This PDF file includes:

Figs. S1 to S13
Tables S1 to S4
References

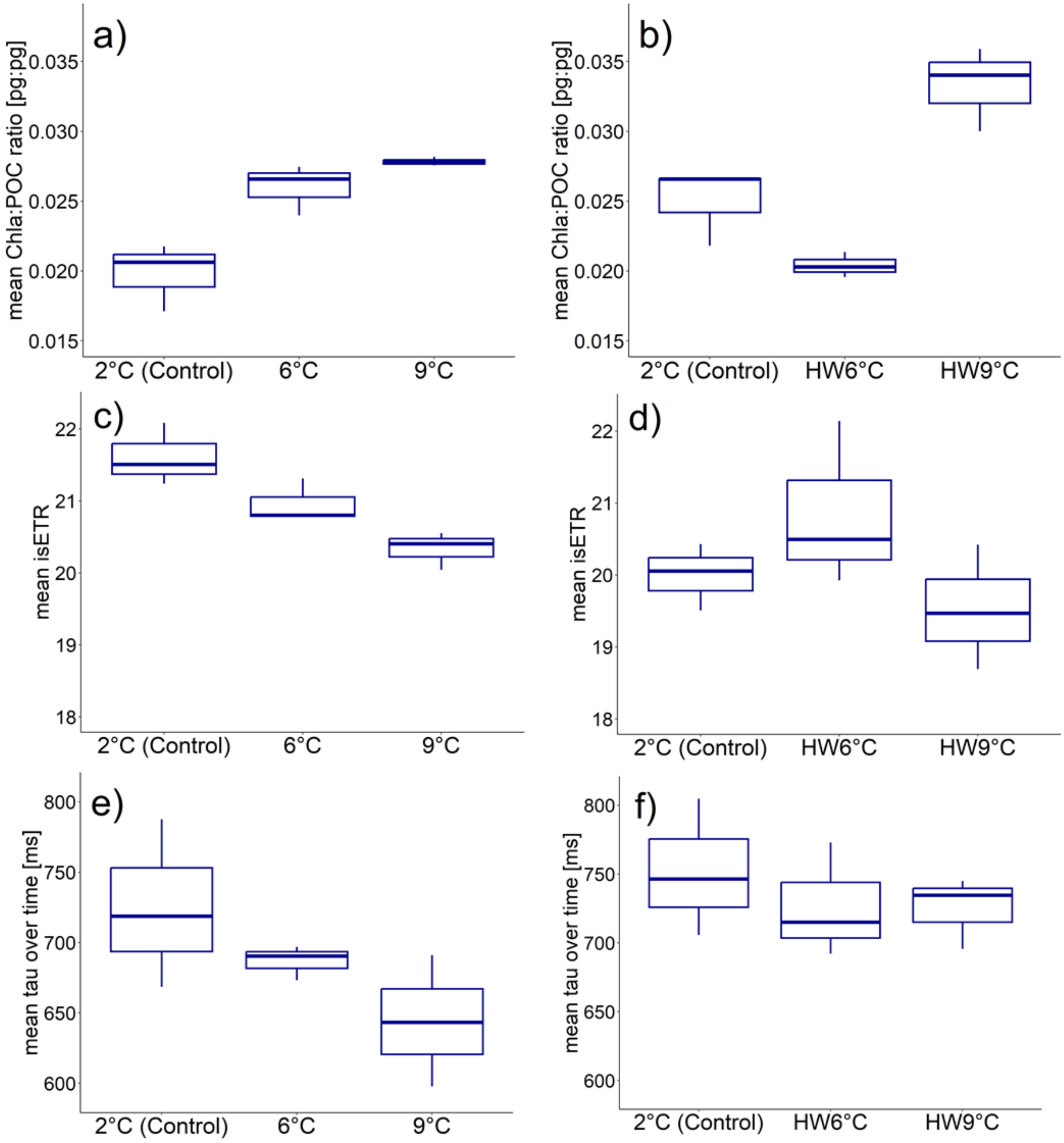

Figure S1 Weighted means over time of additional parameters

Weighted means over time of additional physiological parameters: a) + b) Chl a:POC ratio, c) + d) relative electron transport rate at in-situ/experimental light (isETR), e) + f) re-oxidation time at PSII (tau), left graphs: stable temperature treatments up to day 16; right graphs: heatwave treatments and stable 2°C up to day 20.

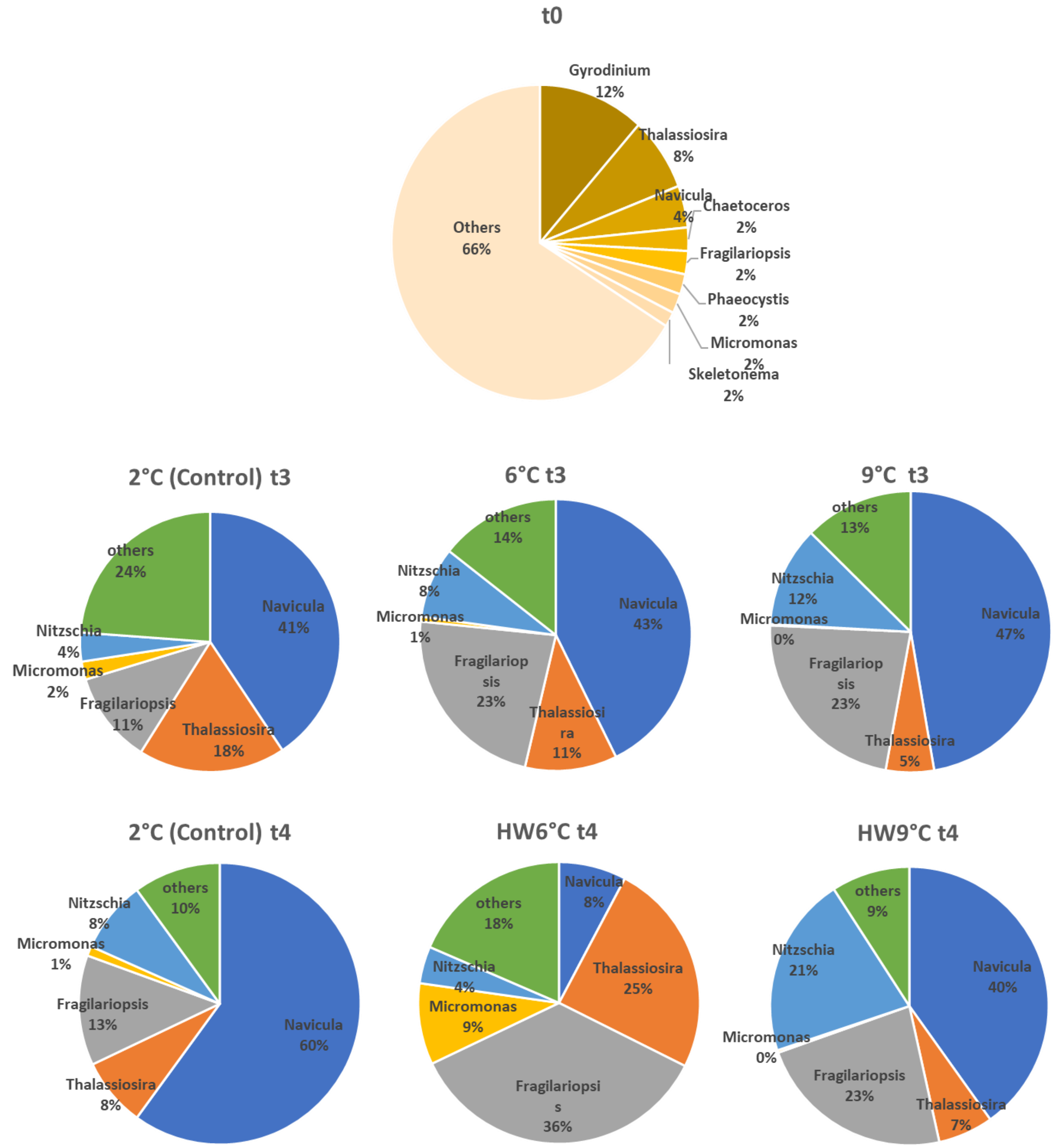

Figure S2: Relative composition of top 5 genera by treatment
Relative composition of top 5 genera by treatment at the initial and final timepoint (t0: top 8 genera). Numbers describe relative abundance of assigned ASVs in percent.

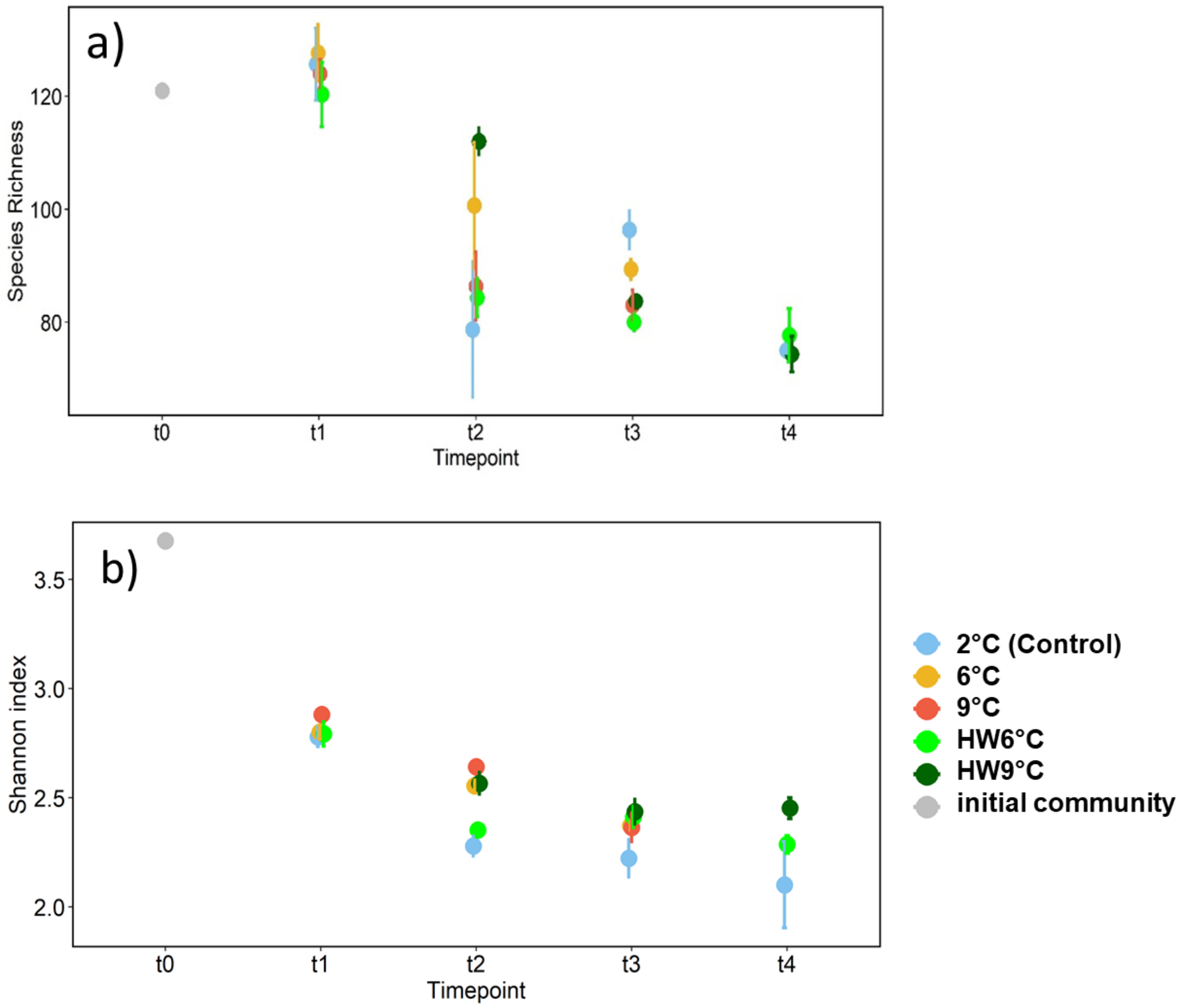

Figure S3 Alpha diversity by species richness (a) and shannon-index (b) of the 5 treatments over time along with the initial community (grey). Diversity and composition were assessed from 18S rRNA gene metabarcoding and abundances are thus only meaningful as relatively within samples.

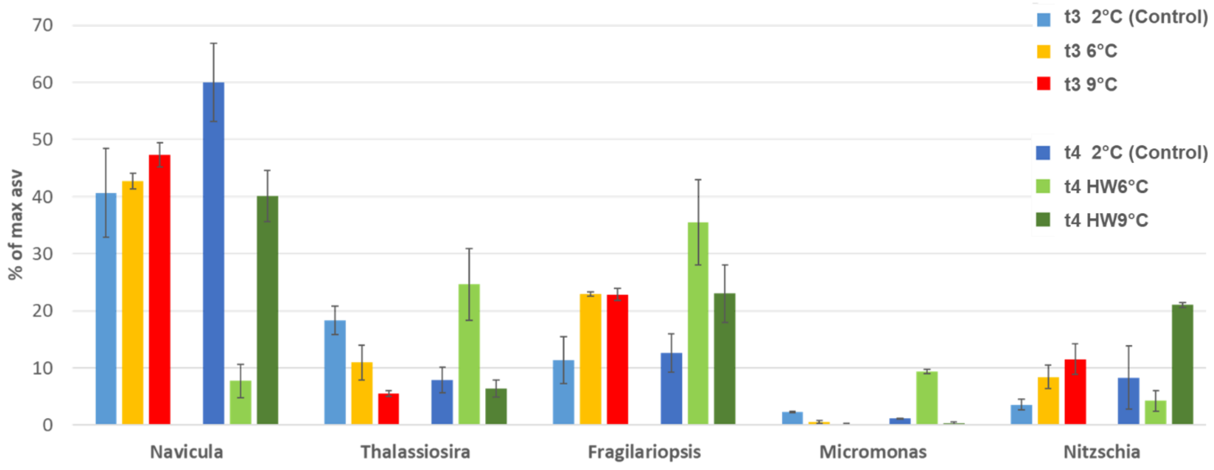

Figure S4 Final relative abundance of top 5 genera by genus

Proportional abundance of top5 dominant genera at the final timepoints of all treatments. The relative abundance of each genus in the different treatments gives an indication for their relative success under the respective conditions. Please note that this data describes relative abundances, and has thus no meaning for the absolute abundance of the respective species.

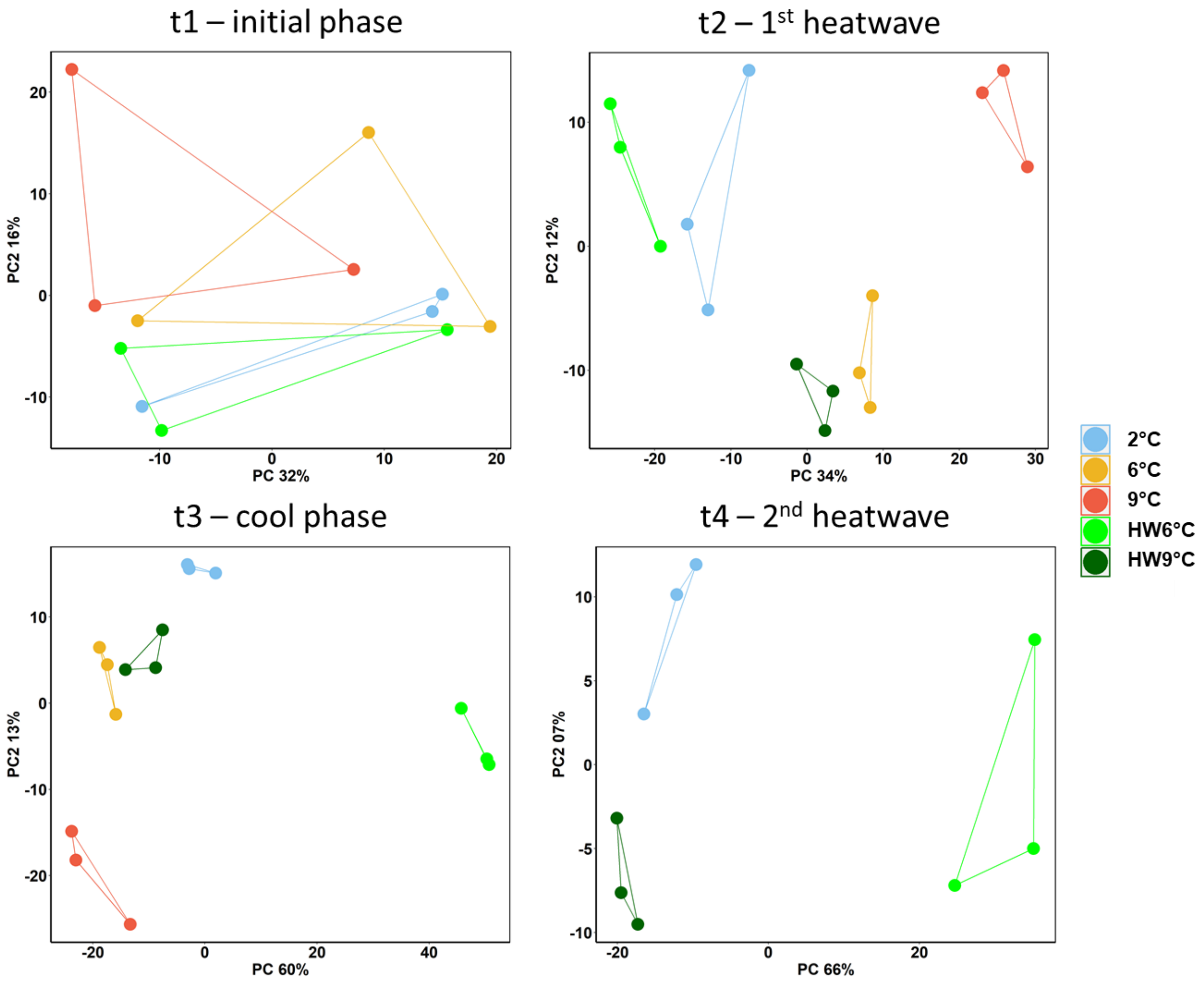

Figure S5 Beta-diversity of species composition over time

PCAs of beta-diversity (Aitchinson distance) between the samples based on relative ASV abundance, color coded by treatment. Permanova results were significant at all timepoints after t1 (Table S3), the most pronounced shift took place in HW6°C at t3, i.e. during the cool-phase after the first heatwave.

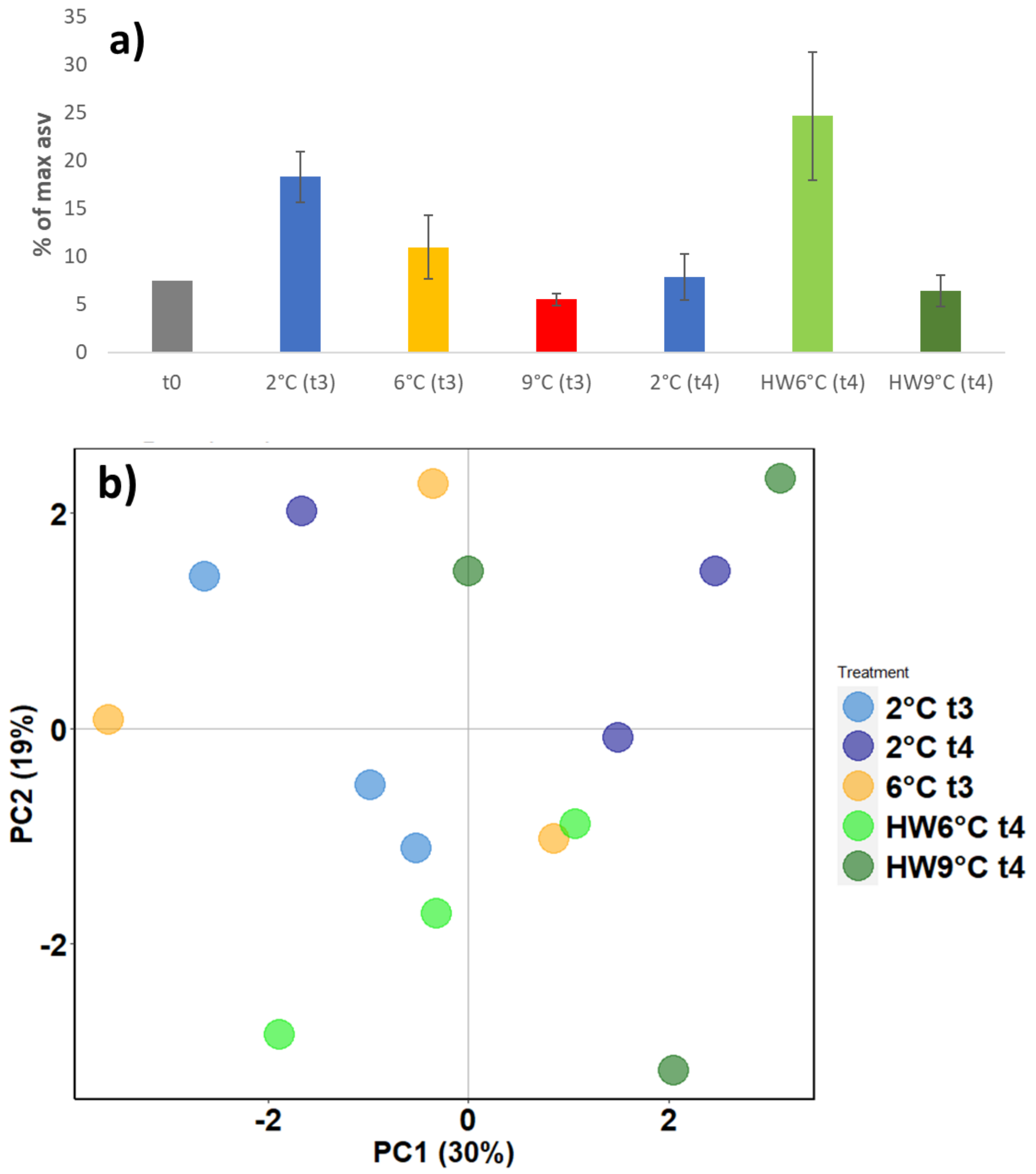

Figure S6 Relative abundance of the genus *Thalassiosira* and PCA of the allelic composition of *T.hyalina* populations

a) Percentage of ASVs assigned to the genus *Thalassiosira* in the initial sample and at the final timepoint of all treatments. b) PCA of the allelic composition of the *T. hyalina* populations at the final timepoints of all treatments analyzed by primer ThKF3. 9°C is missing because the analysis yielded no results here anymore, likely because the species went extinct.

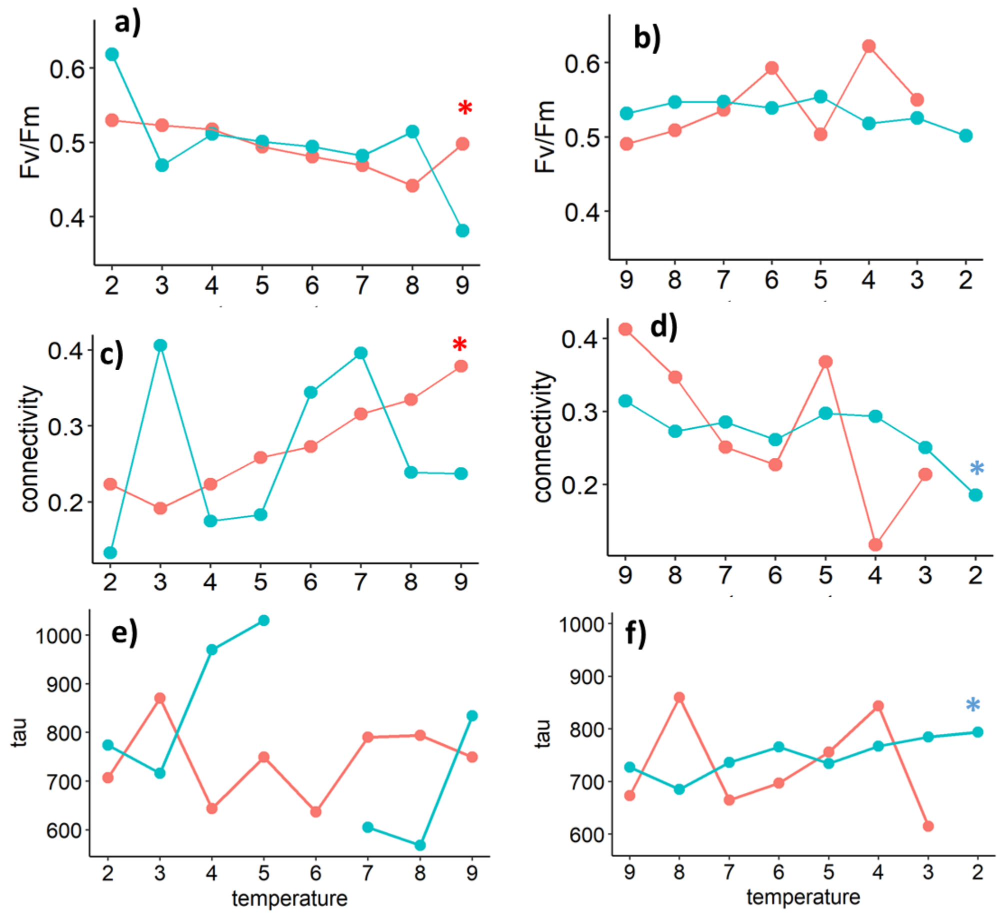

Figure S7 Photophysiological responses of HW9°C assemblages during warming and cooling ramps

Photophysiological response of HW9°C assemblages during warming (left) and cooling (right) ramps (1°C/h). Ramps of the first heatwave are shown in red, ramps of the second heatwave in turquoise. Colored asterisks above the final point mark significant slopes over the entire ramp as tested by linear modes ($p>0.05$). a) + b) Fv/Fm, c) + d) connectivity of photosystems, e) + f) re-oxidation time at PSII (tau).

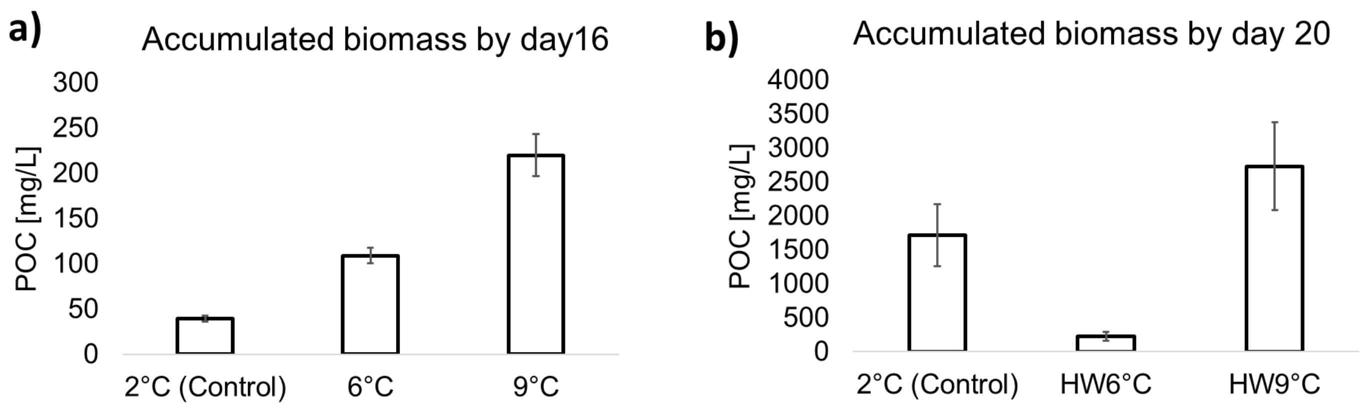

Figure S8 Theoretical stocks calculation

Theoretical accumulated phytoplankton biomass (as particulate organic carbon) without loss terms based on C-specific growth rate after a) 16 days (i.e. 14 warm days) and b) 20 days (i.e. 2x 5 warm days).

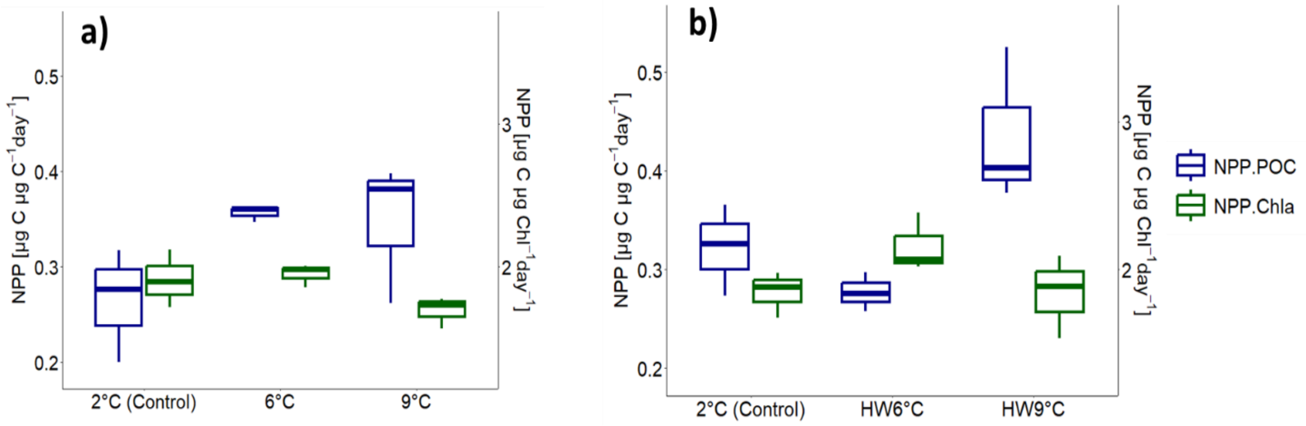

Figure S9 Weighted means of net primary production (NPP) normalized to POC as well as to Chl a

Weighted means over time of net primary production (NPP) normalized to POC (blue) as well as to Chl a (green) for stable temperature (a) and heatwave treatments (b). Please note the fundamentally different trends, which are largely due to the shifts in Chl a:POC ratios of the communities in the different treatments (see Figure S1).

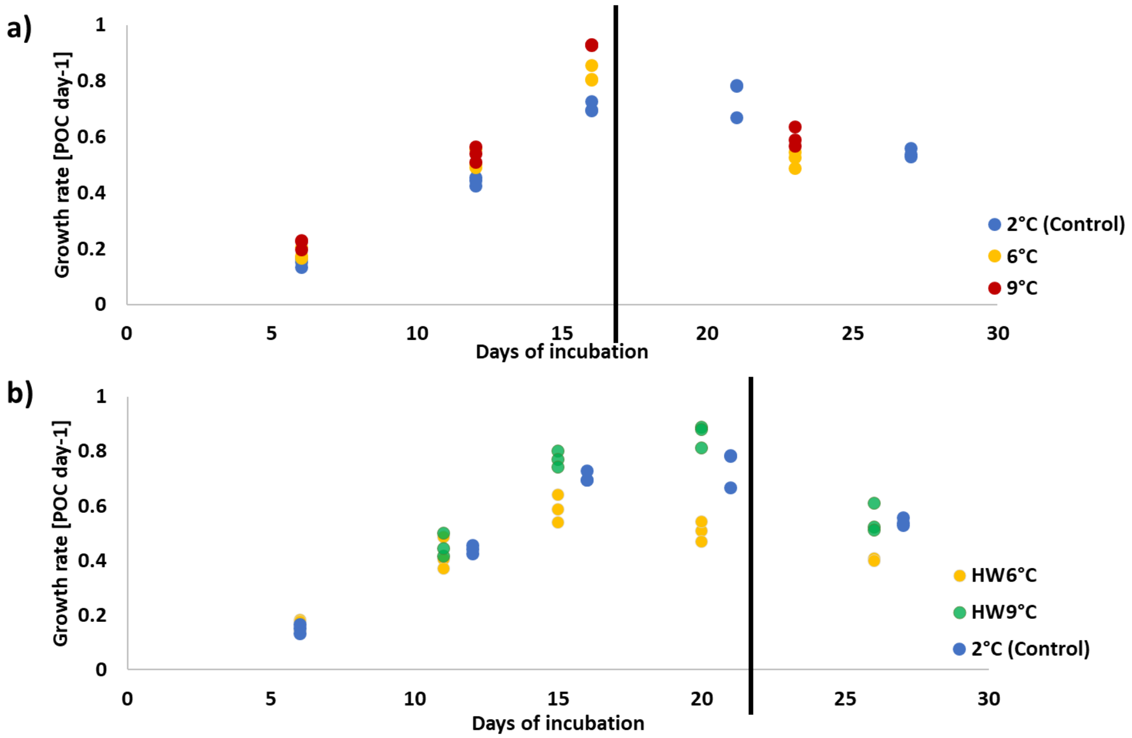

Figure S10 Growth rates including the last timepoint after biofilm removal

Growth rates over the course of the experiment including the last timepoint after biofilm removal (black line), in the stable temperature treatments (a) and the heatwave treatments (b). The measurements after this timepoint were removed from analysis because communities of all treatments showed reduced growth and productivity afterwards.

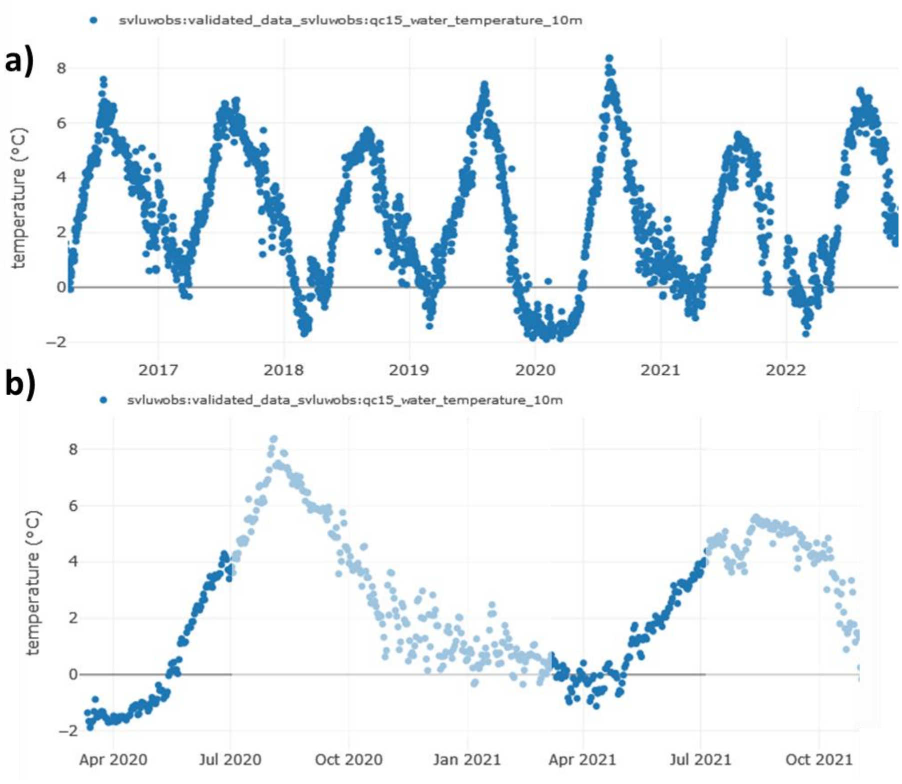

Figure S11: Thermal history of phytoplankton spring bloom community in Konsfjorden, Svalbard, Norway.

a) year-round temperature data from Kongsfjorden 2016-2023 b) example of a zoom-in on temperatures 2020 – 2021 with timeframes of the usual spring bloom highlighted. The summer bloom is usually dominated by different species than the one in spring (see Assmy et al. (ref 71)). Data was taken from a coastal sensor station and is freely available under https://dashboard.awi.de/?dashboard=2847

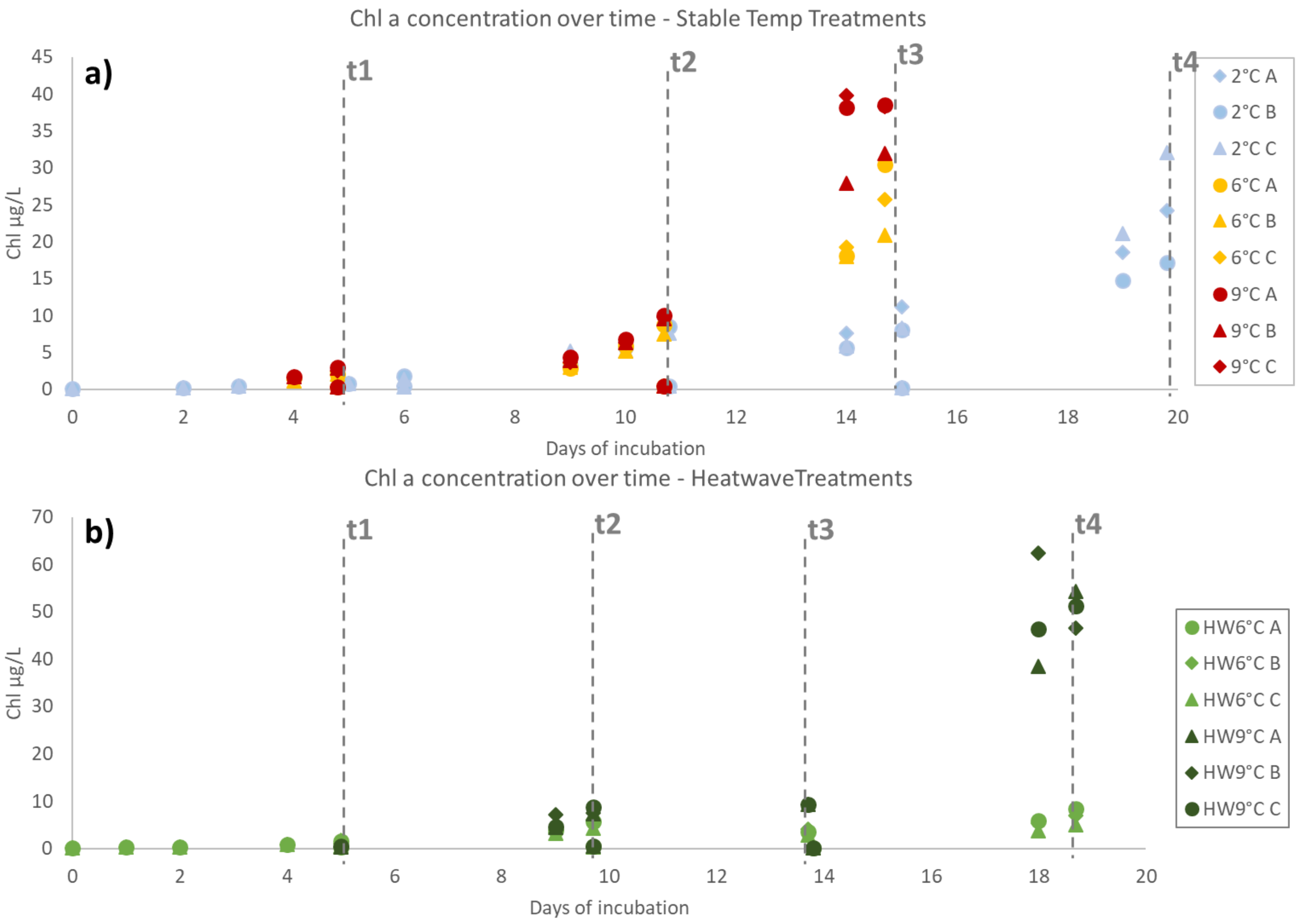

Figure S12: Chl a development throughout the experiment

Phytoplankton biomass dynamics approximated by Chl *a* throughout the experiment in the stable temperature treatments (a) and the heatwave treatments (b). Sampling for all measured parameters and dilutions to ~2µg Chl *a*/L took place at 3-4 timepoints after sampling (t1-t4).

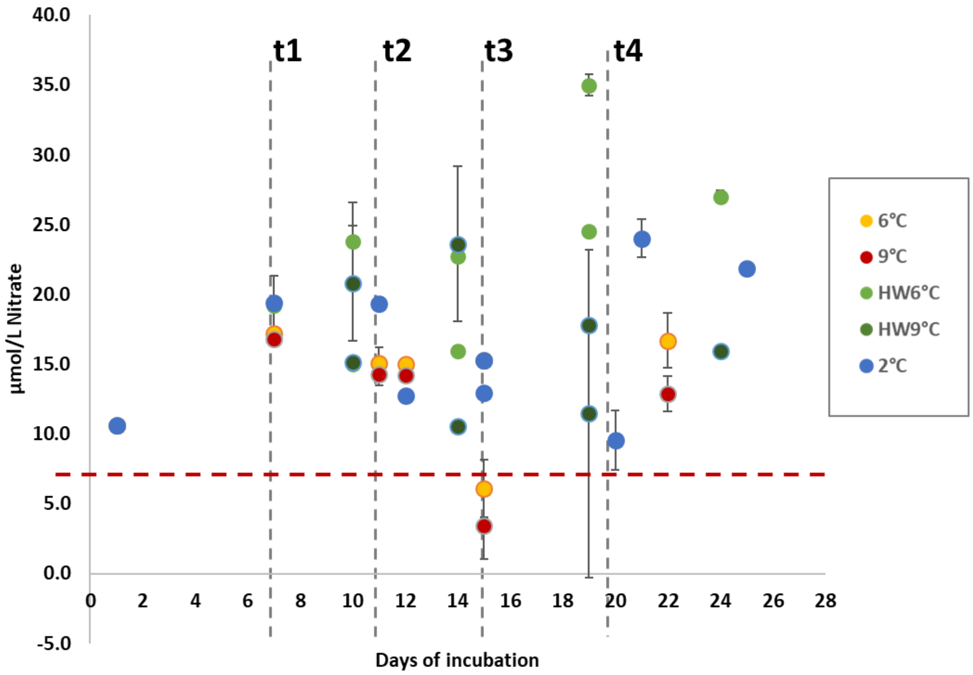

Figure S13: Nitrate concentrations throughout the experiment. Vertical lines mark dilution timepoints, red horizontal line marks 8 µM nitrate as approximate threshold for potential nutrient limitation. Please note that the y-axis is scaled from 0-40 µmol/L and thus excludes excessively added nitrate (to ~60µmol/L) at the initial timepoint in favor of higher resolution of low nitrate concentrations.

Table S1 Summary of statistical results for time-integrated data (linear mixed models)
For statistical testing we used linear mixed-effect models (lmer, lme4 package vers. 1.1.3) on data from all timepoints, with treatment as fixed effects and time and replicate as random effects. A null-model was run on the same data without time as fixed effect. The treatment was reported as having a significant effect if the comparison of the two models (ANOVA) yielded a significant result and $\chi 2$ and p-value were reported. Post-hoc tests were performed using pairwise comparisons of estimated marginal means (emmeans-package, vers. 1.8.7) and Bonferroni correction was applied to control for multiple testing. Model assumptions of linearity and homoscedasticity were verified for each dataset.
Signifiance codes: '***' <0.001 '**' <0.01 '*' <0.05

Dataset	Parameter	Model	Treatments tested	df	χ^2	p-value	Sign. level
Stable temp. treatments (t3)	Growth	ANOVA of lmer (treatments) vs. lmer(null)	2°C, 6°C, 9°C	2	30.2	0.0000003	***
		Post-hoc	6°C vs 2°C	23.8	-	0.002	***
		Post-hoc	9°C vs 2°C	23.8	-	0.000001	***
Stable temp. treatments (t3)	NPP	ANOVA of lmer (treatments) vs. lmer(null)	2°C, 6°C, 9°C	2	14.8	0.0006	***
		Post-hoc	6°C vs 2°C	22.8		0.138	
		Post-hoc	9°C vs 2°C	23.1		0.690	
Stable temp. treatments (t3)	GPP	ANOVA of lmer (treatments) vs. lmer(null)	2°C, 6°C, 9°C	2	3.3	0.190	
		Post-hoc	6°C vs 2°C	18.2		1.0	
		Post-hoc	9°C vs 2°C	18.8		0.670	
Stable temp. treatments (t3)	Dark resp.	ANOVA of lmer (treatments) vs. lmer(null)	2°C, 6°C, 9°C	2	20.2	0.00004	***
		Post-hoc	6°C vs 2°C	18.1		0.005	**
		Post-hoc	9°C vs 2°C	18.3		0.0001	***
Stable temp. treatments (t3)	Chl:POC	ANOVA of lmer (treatments) vs. lmer(null)	2°C, 6°C, 9°C	2	11.915	0.003	**
		Post-hoc	6°C vs 2°C	23.8		0.063	*
		Post-hoc	9°C vs 2°C	23.8		0.014	**
Stable temp. treatments (t3)	isETR	ANOVA of lmer (treatments) vs. lmer(null)	2°C, 6°C, 9°C	2	4.9	0.026	*
		Post-hoc	6°C vs 2°C	23.8		1.0	
		Post-hoc	9°C vs 2°C	23.8		0.193	
Heatwaves and stable 2°C (t4)	Growth	ANOVA of lmer (treatments) vs. lmer(null)	2°C, HW6°C, HW9°C	2	14.8	0.0006	***
		Post-hoc	HW6°C vs 2°C	34.1		0.138	
		Post-hoc	HW9°C vs 2°C	34.1		0.690	
Heatwaves and stable 2°C (t4)	NPP	ANOVA of lmer (treatments) vs. lmer(null)	2°C, HW6°C, HW9°C	2	2.7	0.260	
		Post-hoc	HW6°C vs 2°C	34.6		1.0	
		Post-hoc	HW9°C vs 2°C	33.5		1.0	

Heatwaves and stable 2°C (t4)	GPP	ANOVA of lmer (treatments) vs. lmer(null)	2°C, HW6°C, HW9°C	2	14.0	0.00095	***
		Post-hoc	HW6°C vs 2°C	27.1		0.343	
		Post-hoc	HW9°C vs 2°C	27.1		0.276	
Heatwaves and stable 2°C (t4)	Dark resp.	ANOVA of lmer (treatments) vs. lmer(null)	2°C, HW6°C, HW9°C	2	2.6	0.268	
		Post-hoc	HW6°C vs 2°C	24.5		1.0	
		Post-hoc	HW9°C vs 2°C	24.9		0.880	
Heatwaves and stable 2°C (t4)	Chl:POC	ANOVA of lmer (treatments) vs. lmer(null)	2°C, HW6°C, HW9°C	2	11.9	0.003	**
		Post-hoc	HW6°C vs 2°C	32.1		0.106	
		Post-hoc	HW9°C vs 2°C	32.1		1.0	
Heatwaves and stable 2°C (t4)	isETR	ANOVA of lmer (treatments) vs. lmer(null)	2°C, HW6°C, HW9°C	2	0.8	0.664	

Table S2 Summary of statistical results for time-resolved data

For the time-resolved data, we used one-way-ANOVAs to identify differences between treatments at specific timepoints in question or between timepoints within a treatment (e.g. in response to temperature change). For a further analysis of the separate treatments, we used Tukey post-hoc tests. Furthermore, we used linear models (lm) for regression analysis over several timepoints. Also here, model assumptions of linearity and homoscedasticity were verified in each case. Significance codes: ‘***’ <0.001 ‘**’ <0.01 ‘*’ <0.05

Dataset	Parameter	Test/Model	slope	adj. r^2 / F	df	p-value	Sign. level
2°C treatment (t1-t3)	Growth	Linear model (lm)	0.055	0.99	-	$4.91e^{-08}$	***
6°C treatment (t1-t3)	Growth	Linear model (lm)	0.063	0.99	-	$1.52e^{-07}$	***
9°C treatment (t1-t3)	Growth	Linear model (lm)	0.070	0.96	-	$7.74e^{-09}$	***
2°C treatment (t1-t3)	NPP	Linear model (lm)	0.012	0.22	-	0.112	
6°C treatment (t1-t3)	NPP	Linear model (lm)	0.029	0.93	-	0.00002	***
9°C treatment (t1-t3)	NPP	Linear model (lm)	0.051	0.89	-	0.0003	***
2°C treatment (t1-t3)	Resp	Linear model (lm)	-	0.11	-	0.169	
6°C treatment (t1-t3)	Resp	Linear model (lm)	-	0.35	-	0.128	
9°C treatment (t1-t3)	Resp	Linear model (lm)	-	0.73	-	0.009	
Stable temp. treatments (t2)	Resp	ANOVA	-	15.21	2	0.003	**
Stable temp. treatments (t3)	Resp	ANOVA	-	7.07	2	0.021	*
Heatwaves and stable 2°C (t2)	Growth	ANOVA	-	0.47	2	0.646	
Heatwaves and stable 2°C (t3)	Growth	ANOVA	-	20.11	2	0.002	**
		Tukey Post-hoc t4: HW6°C vs 2°C				0.016	*
		Tukey Post-hoc t4: HW9°C vs 2°C				0.142	
Heatwaves and stable 2°C (t4)	Growth	ANOVA	-	38.55	2	0.0004	***
		Tukey Post-hoc t4: HW6°C vs 2°C				0.003	**
		Tukey Post-hoc t4: HW9°C vs 2°C				0.070	
Heatwaves and stable 2°C (t2)	NPP	ANOVA	-	7.905	2	0.021	*
		Tukey Post-hoc t4: HW6°C vs 2°C				0.312	
		Tukey Post-hoc t4: HW6°C vs 2°C				0.017	*
Heatwaves and stable 2°C (t3)	NPP	ANOVA	-	3.068	2	0.121	
Heatwaves and stable 2°C (t4)	NPP	ANOVA	-	4.883	2	0.092	

Table S3 Summary of statistical results for beta-diversity of relative compositional data
Following (Gloor et al. (ref 70)), beta-diversity was estimated through pairwise dissimilarity matrices using Aitchison-distances, i.e. the Euclidean distance of centered-log ratio (clr) transformed raw data, and was visualized through principal component analysis (PCA). Treatment differences were tested by permanova analysis.
Significance codes: '***' <0.001 '**' <0.01 '*' <0.05

Dataset	Timepoint	Test	df	r^2	F	p	Sign. level
Beta-diversity: all treatments	t1	Permanova	3	0.28	1.05	0.367	
Beta-diversity: All treatments	T2	Permanova	4	0.51	2.58	0.0001	***
Beta-diversity: All treatments	T3	Permanova	4	0.67	5.05	0.0001	***
Beta-diversity: 2°C, HW6°C, HW9°C	T4	Permanova	2	0.63	5.00	0.005	**
Beta-diversity: All treatments	Tfin (t3 for 2°C, 6°C, 9°C+t4 for 2°C, HW6°C, HW9°C)	Permanova	5	0.61	3.83	0.0001	***
Beta-diversity: 2°C, 6°C, 9°C	Stable treatments tfin (t3)	Permanova	2	0.52276	3.2862	0.0031	**
Beta-diversity: 2°C, HW6°C, HW9°C	Heatwave treatments tfin (t4)	Permanova	2	0.62505	5.0011	0.0037	**

Table S4 Sequencing statistics for all metabarcoding samples after each filtering step

Sequencing statistics for all experimental samples by 18S rRNA metabarcoding after each filtering step and the ratio of final reads to raw reads.

sample	raw	quality-filtered	denoised - forward	denoised-reverse	merged	chimera-filtered	ratio filtered/raw
2C-A-t1	78306	56695	56535	56607	53816	53151	0.68
2C-A-t2	36536	12361	12318	12338	11279	11061	0.30
2C-A-t3	232967	180807	180608	180657	171085	166069	0.71
2C-A-t4	138167	103705	103594	103579	98349	95487	0.69
2C-B-t1	81095	59704	59487	59495	56382	55607	0.69
2C-B-t2	119966	89043	88985	88999	84058	82322	0.69
2C-B-t3	182040	137746	137634	137644	129328	125854	0.69
2C-B-t4	101851	77063	76977	77001	73532	72059	0.71
2C-C-t1	220681	153072	152778	152912	144589	142548	0.65
2C-C-t2	86668	66905	66841	66858	63474	62117	0.72
2C-C-t3	182482	140501	140373	140396	132232	128626	0.70
2C-C-t4	110375	79885	79750	79801	75866	74301	0.67
6C-A-t1	949464	688601	688023	688064	650977	642179	0.68
6C-A-t2	104663	69641	69549	69582	65998	65661	0.63
6C-A-t3	174850	131807	131691	131691	124477	121979	0.70
6C-B-t1	83590	59230	59074	59124	56349	55639	0.67
6C-B-t2	1357760	802339	801782	801930	737456	723894	0.53
6C-B-t3	210762	161929	161776	161795	152160	147654	0.70
6C-C-t1	112223	79204	79058	79105	74786	74017	0.66
6C-C-t2	111362	83115	83066	83071	79085	77743	0.70
6C-C-t3	211130	157442	157290	157264	144843	140402	0.67
9C-A-t1	935652	644696	644284	644286	610449	607395	0.65
9C-A-t2	115580	77167	77111	77113	72856	71978	0.62
9C-A-t3	207631	156863	156294	156396	146657	143115	0.69
9C-B-t1	108557	76191	75884	75963	72083	71183	0.66
9C-B-t2	222366	166019	165905	165869	156407	154537	0.69
9C-B-t3	231276	174408	174113	174231	164269	160556	0.69
9C-C-t1	93634	64831	64667	64679	61770	61066	0.65

9C-C-t2	86612	62826	62747	62752	57972	57491	0.66
9C-C-t3	181353	137712	137577	137578	129779	127044	0.70
HW6C-A-t1	91356	65400	65233	65280	61997	61256	0.67
HW6C-A-t2	117746	79714	79631	79668	75163	74126	0.63
HW6C-A-t3	96681	72282	72238	72247	68585	67814	0.70
HW6C-A-t4	132846	102643	102533	102566	96550	94969	0.71
HW6C-B-t1	60814	36207	36020	36077	33515	33141	0.54
HW6C-B-t2	123493	77377	77285	77299	72991	71776	0.58
HW6C-B-t3	128974	100980	100895	100903	95702	94098	0.73
HW6C-B-t4	106491	80939	80887	80860	75835	74575	0.70
HW6C-C-t1	187239	139586	139294	139390	131848	129732	0.69
HW6C-C-t2	106223	71682	71619	71609	67840	66891	0.63
HW6C-C-t3	146808	108604	108535	108531	102517	100333	0.68
HW6C-C-t4	100437	76445	76362	76394	72714	71796	0.71
HW9C-A-t2	332393	231270	231082	231142	217319	213760	0.64
HW9C-A-t3	94583	73134	73083	73068	69301	68301	0.72
HW9C-A-t4	223861	169566	169405	169348	160146	155251	0.69
HW9C-B-t2	211986	141480	141378	141359	133750	131289	0.62
HW9C-B-t3	110746	83830	83748	83778	79351	77895	0.70
HW9C-B-t4	175275	125948	125814	125842	119249	115969	0.66
HW9C-C-t2	271028	172069	171924	171928	161817	158010	0.58
HW9C-C-t3	114895	89678	89619	89621	85353	84058	0.73
HW9C-C-t4	209055	156595	156325	156371	148011	143885	0.69
ALL	10403841					6781071	0.65

REFERENCES AND NOTES

1. E. C. J. Oliver, M. T. Burrows, M. G. Donat, A. Sen Gupta, L. V. Alexander, S. E. Perkins-Kirkpatrick, J. A. Benthuysen, A. J. Hobday, N. J. Holbrook, P. J. Moore, M. S. Thomsen, T. Wernberg, D. A. Smale, Projected marine heatwaves in the 21st century and the potential for ecological impact. *Front. Mar. Science* **6**, 364 (2019).

2. S. Cooley, D. Schoeman, L. Bopp, P. Boyd, S. Donner, S. Ito, W. Kiessling, P. Martinetto, E. Ojea, M.-F. Racault, B. Rost, M. Skern-Mauritzen, D. Y. Ghebrehiwet, J. D. Bell, J. Blanchard, J. Bolin, W. W. Cheung, A. Cisneros-Montemayor, S. Dupont, S. Dutkiewicz, T. Frölicher, J. D. Gaitán-Espitia, J. G. Molinos, H. Gurney-Smith, S. Henson, M. Hidalgo, E. Holland, R. Kopp, R. Kordas, L. Kwiatkowski, N. Le Bris, S. E. Lluch-Cota, C. Logan, F. C. Mark, Y. Mgaya, C. Moloney, N. P. Muñoz Sevilla, G. Randin, N. B. Raja, A. Rajkaran, A. Richardson, S. Roe, R. Ruiz Diaz, D. Salili, J. B. Sallée, K. Scales, M. Scobie, C. T. Simmons, O. Torres, A. Yool, "Chapter 3: Oceans and Coastal Ecosystems and their Services," in Climate Change 2022: Impacts, adaptation and vulnerability. Contribution of the WGII to the 6th assessment report of the intergovernmental panel on climate change No. IPCC AR6 WGII (IPCC, 2022).

3. L. A. Arteaga, C. S. Rousseaux, Impact of Pacific Ocean heatwaves on phytoplankton community composition. *Commun. Biol.* **6**, 263 (2023).

4. B. Huang, Z. Wang, X. Yin, A. Arguez, G. Graham, C. Liu, T. Smith, H. M. Zhang, Prolonged marine heatwaves in the Arctic: 1982−2020. *Geophys. Res. Lett.* **48**, e2021GL095590 (2021).

5. T. L. Frölicher, E. M. Fischer, N. Gruber, Marine heatwaves under global warming. *Nature* **560**, 360–364 (2018).

6. A. Barkhordarian, D. M. Nielsen, D. Olonscheck, J. Baehr, Arctic marine heatwaves forced by greenhouse gases and triggered by abrupt sea-ice melt. *Commun. Earth Environ.* **5**, 57 (2024).

7. T. L. Frölicher, C. Laufkötter, Emerging risks from marine heat waves. *Nat. Commun.* **9**, 650 (2018).

8. A. J. Constable, S. Harper, J. Dawson, T. Mustonen, D. Piepenburg, B. Rost, S. Bokhorst, J. Boike, A. Cunsolo, C. Derksen, Cross-chapter paper 6: Polar regions, in *Climate Change 2022: Impacts, Adaptation and Vulnerability* (IPCC, 2022).

9. M. P. Latorre, C. M. Iachetti, I. R. Schloss, J. Antoni, A. Malits, F. de la Rosa, M. De Troch, M. D. Garcia, X. Flores-Melo, S. I. Romero, M. N. Gil, M. Hernando, Summer heatwaves affect coastal Antarctic plankton metabolism and community structure. *J. Exp. Mar. Biol. Ecol.* **567**, 151926 (2023).

10. L. M. Cavole, A. M. Demko, R. E. Diner, A. Giddings, I. Koester, C. M. L. S. Pagniello, M.-L. Paulsen, A. Ramirez-Valdez, S. M. Schwenck, N. K. Yen, M. E. Zill, P. J. S. Franks, Biological impacts of the 2013–2015 warm-water anomaly in the Northeast Pacific: Winners, losers, and the future. *Oceanography* **29**, 273–285 (2016).

11. J. E. Walsh, R. L. Thoman, U. S. Bhatt, P. A. Bieniek, B. Brettschneider, M. Brubaker, S. Danielson, R. Lader, F. Fetterer, K. Holderied, The high latitude marine heat wave of 2016 and its impacts on Alaska. *Bull. Am. Meteorol. Soc.* **99**, S39–S43 (2018).

12. K. E. Mills, A. J. Pershing, C. J. Brown, Y. Chen, F.-S. Chiang, D. S. Holland, S. Lehuta, J. A. Nye, J. C. Sun, A. C. Thomas, R. A. Wahle, Fisheries management in a changing climate: Lessons from the 2012 ocean heat wave in the Northwest Atlantic. *Oceanography* **26**, 191–195 (2013).

13. R. M. McCabe, B. M. Hickey, R. M. Kudela, K. A. Lefebvre, N. G. Adams, B. D. Bill, F. M. D. Gulland, R. E. Thomson, W. P. Cochlan, V. L. Trainer, An unprecedented coastwide toxic algal bloom linked to anomalous ocean conditions. *Geophys. Res. Lett.* **43**, 10366–310376 (2016).

14. D. M. Anderson, E. Fachon, R. S. Pickart, P. Lin, A. D. Fischer, M. L. Richlen, V. Uva, M. L. Brosnahan, L. McRaven, F. Bahr, K. Lefebvre, J. M. Grebmeier, S. L. Danielson, Y. Lyu, Y. Fukai, Evidence for massive and recurrent toxic blooms of Alexandrium catenella in the Alaskan Arctic. *Proc. Natl. Acad. Sci. U.S.A.* **118**, e2107387118 (2021).

15. D. A. Smale, T. Wernberg, E. C. J. Oliver, M. Thomsen, B. P. Harvey, S. C. Straub, M. T. Burrows, L. V. Alexander, J. A. Benthuysen, M. G. Donat, M. Feng, A. J. Hobday, N. J. Holbrook, S. E. Perkins-

Kirkpatrick, H. A. Scannell, A. Sen Gupta, B. L. Payne, P. J. Moore, Marine heatwaves threaten global biodiversity and the provision of ecosystem services. *Nat. Clim. Change* **9**, 306–312 (2019).

16. J. E. Bissinger, D. J. Montagnes, J. Sharples, D. Atkinson, Predicting marine phytoplankton maximum growth rates from temperature: Improving on the Eppley curve using quantile regression. *Limnol. Oceanogr.* **53**, 487–493 (2008).

17. R. W. Eppley, Temperature and phytoplankton growth in the sea. *Fish. Bull.* **70**, 1063–1085 (1972).

18. M. J. Angilletta, Thermal adaptation: A theoretical and empirical synthesis (Oxford Univ. Press, 2009).

19. M. J. Cabrerizo, E. Marañón, Temperature fluctuations in a warmer environment: Impacts on microbial plankton. *Fac. Rev.* **10**, 9 (2021).

20. X. Wang, F. Fu, P. Qu, J. D. Kling, H. Jiang, Y. Gao, D. A. Hutchins, How will the key marine calcifier Emiliania huxleyi respond to a warmer and more thermally variable ocean? *Biogeosciences* **16**, 4393–4409 (2019).

21. J. R. Bernhardt, J. M. Sunday, P. L. Thompson, M. I. O'Connor, Nonlinear averaging of thermal experience predicts population growth rates in a thermally variable environment. *Proc. Natl. Acad. Sci. U.S.A.* **285**, 20181076 (2018).

22. P. Qu, F. X. Fu, J. D. Kling, M. Huh, X. Wang, D. A. Hutchins, Distinct responses of the nitrogen-fixing marine cyanobacterium Trichodesmium to a thermally variable environment as a function of phosphorus availability. *Front. Microbiol.* **10**, 1282 (2019).

23. C. E. Schaum, A. Buckling, N. Smirnoff, D. J. Studholme, G. Yvon-Durocher, Environmental fluctuations accelerate molecular evolution of thermal tolerance in a marine diatom. *Nat. Commun.* **9**, 1719 (2018).

24. P. Siegel, K. G. Baker, E. Low-Décarie, R. J. Geider, Phytoplankton competition and resilience under fluctuating temperature. *Ecol. Evol.* **13**, e9851 (2023).

25. H. Hayashida, R. J. Matear, P. G. Strutton, Background nutrient concentration determines phytoplankton bloom response to marine heatwaves. *Glob. Chang. Biol.* **26**, 4800–4811 (2020).

26. K. M. Noh, H.-G. Lim, J.-S. Kug, Global chlorophyll responses to marine heatwaves in satellite ocean color. *Environ. Res. Lett.* **17**, 064034 (2022).

27. A. Sen Gupta, M. Thomsen, J. A. Benthuysen, A. J. Hobday, E. Oliver, L. V. Alexander, M. T. Burrows, M. G. Donat, M. Feng, N. J. Holbrook, S. Perkins-Kirkpatrick, P. J. Moore, R. R. Rodrigues, H. A. Scannell, A. S. Taschetto, C. C. Ummenhofer, T. Wernberg, D. A. Smale, Drivers and impacts of the most extreme marine heatwave events. *Sci. Rep.* **10**, 19359 (2020).

28. W. K. Zhan, Y. Zhang, Q. Y. He, H. G. Zhan, Shifting responses of phytoplankton to atmospheric and oceanic forcing in a prolonged marine heatwave. *Limnol. Oceanogr.* **68**, 1821–1834 (2023).

29. S. Montie, M. S. Thomsen, W. Rack, P. A. Broady, Extreme summer marine heatwaves increase chlorophyllain the Southern Ocean. *Antarct. Sci.* **32**, 508–509 (2020).

30. E. Feijão, C. Gameiro, M. Franzitta, B. Duarte, I. Caçador, M. T. Cabrita, A. R. Matos, Heat wave impacts on the model diatom Phaeodactylum tricornutum: Searching for photochemical and fatty acid biomarkers of thermal stress. *Ecol. Indicators* **95**, 1026–1037 (2018).

31. T. Samuels, T. A. Rynearson, S. Collins, Surviving heatwaves: Thermal experience predicts life and death in a Southern Ocean diatom. *Front. Mar. Sci* **8**, 343 (2021).

32. T. Soulié, F. Vidussi, S. Mas, B. Mostajir, Functional stability of a coastal Mediterranean plankton community during an experimental marine heatwave. *Front. Mar. Sci.* **9**, 831496 (2022).

33. N. Filiz, U. Işkın, M. Beklioğlu, B. Öğlü, Y. Cao, T. A. Davidson, M. Søndergaard, T. L. Lauridsen, E. Jeppesen, Phytoplankton community response to nutrients, temperatures, and a heat wave in shallow lakes: An experimental approach. *Water* **12**, 3394 (2020).

34. J. D. Kling, M. D. Lee, F. Fu, M. D. Phan, X. Wang, P. Qu, D. A. Hutchins, Transient exposure to novel high temperatures reshapes coastal phytoplankton communities. *ISME J.* **14**, 413–424 (2020).

35. M. Remy, H. Hillebrand, S. Flöder, Stability of marine phytoplankton communities facing stress related to global change: Interactive effects of heat waves and turbidity. *J. Exp. Mar. Biol. Ecol.* **497**, 219–229 (2017).

36. C. T. Kremer, M. K. Thomas, E. Litchman, Temperature- and size-scaling of phytoplankton population growth rates: Reconciling the Eppley curve and the metabolic theory of ecology. *Limnol. Oceanogr.* **62**, 1658–1670 (2017).

37. I. W. Bishop, S. I. Anderson, S. Collins, T. A. Rynearson, Thermal trait variation may buffer Southern Ocean phytoplankton from anthropogenic warming. *Glob. Chang. Biol.* **28**, 5755–5767 (2022).

38. M. K. Thomas, C. T. Kremer, C. A. Klausmeier, E. Litchman, A global pattern of thermal adaptation in marine phytoplankton. *Science* **338**, 1085–1088 (2012).

39. A. Coello-Camba, S. Agustí, Thermal thresholds of phytoplankton growth in polar waters and their consequences for a warming polar ocean. *Front. Mar. Sci.* **4**, 168 (2017).

40. S. Barton, J. Jenkins, A. Buckling, C.-E. Schaum, N. Smirnoff, J. A. Raven, G. Yvon-Durocher, Evolutionary temperature compensation of carbon fixation in marine phytoplankton. *Ecol. Lett.* **23**, 722–733 (2020).

41. D. Bozzato, T. Jakob, C. Wilhelm, Effects of temperature and salinity on respiratory losses and the ratio of photosynthesis to respiration in representative Antarctic phytoplankton species. *PLOS ONE* **14**, e0224101 (2019).

42. K. G. Baker, C. M. Robinson, D. T. Radford, A. S. McInnes, C. Evenhuis, M. A. Doblin, Thermal performance curves of functional traits aid understanding of thermally induced changes in diatom-mediated biogeochemical fluxes. *Front. Mar. Sci.* **3**, 44 (2016).

43. L. Rehder, B. Rost, S. D. Rokitta, Abrupt and acclimation responses to changing temperature elicit divergent physiological effects in the diatom Phaeodactylum tricornutum. *New Phytol.* **239**, 1005–1013 (2023).

44. P. A. Thompson, M.-X. Guo, P. J. Harrison, Effects of variation in temperature. I. On the biochemical composition of eight species of marine phytoplankton. *J. Phycol.* **28**, 481–488 (1992).

45. S. Collins, A. Gardner, Integrating physiological, ecological and evolutionary change: A Price equation approach. *Ecol. Lett.* **12**, 744–757 (2009).

46. C. J. M. Hoppe, N. Schuback, D. Semeniuk, K. Giesbrecht, J. Mol, H. Thomas, M. T. Maldonado, B. Rost, D. E. Varela, P. D. Tortell, Resistance of Arctic phytoplankton to ocean acidification and enhanced irradiance. *Polar Biol.* **41**, 399–413 (2018).

47. K. K. E. Wolf, C. J. M. Hoppe, F. Leese, M. Weiss, B. Rost, S. Neuhaus, T. Gross, N. Kühne, U. John, Revealing environmentally driven population dynamics of an Arctic diatom using a novel microsatellite PoolSeq barcoding approach. *Environ. Microbiol.* **23**, 3809–3824 (2021).

48. K. K. E. Wolf, E. Romanelli, B. Rost, U. John, S. Collins, H. Weigand, C. J. Hoppe, Company matters: The presence of other genotypes alters traits and intraspecific selection in an Arctic diatom under climate change. *Glob. Chang. Biol.* **25**, 2869–2884 (2019).

49. L. I. Seifert, G. Weithoff, M. Vos, Extreme heat changes post-heat wave community reassembly. *Ecol. Evol.* **5**, 2140–2148 (2015).

50. I. E. Huertas, M. Rouco, V. López-Rodas, E. Costas, Warming will affect phytoplankton differently: Evidence through a mechanistic approach. *Proc. R. Soc. B: Biol. Sci.* **278**, 3534–3543 (2011).

51. P. G. Falkowski, J. A. Raven, *Aquatic photosynthesis*. (Princeton University Press, 2013).

52. M. J. Behrenfeld, K. H. Halsey, A. J. Milligan, Evolved physiological responses of phytoplankton to their integrated growth environment. *Philosoph. Trans. R. Soc. B: Biol. Sci.* **363**, 2687–2703 (2008).

53. M. Seifert, C. Nissen, B. Rost, M. Vogt, C. Volker, J. Hauck, Interaction matters: Bottom-up driver interdependencies alter the projected response of phytoplankton communities to climate change. *Glob. Chang. Biol.* **29**, 4234–4258 (2023).

54. C. A. E. McKinstry, R. W. Campbell, K. Holderied, Influence of the 2014–2016 marine heatwave on seasonal zooplankton community structure and abundance in the lower Cook Inlet, Alaska. *Deep-Sea Res. II Top. Stud. Oceanogr.* **195**, 105012 (2022).

55. S. D. Batten, C. Ostle, P. Hélaouët, A. W. Walne, Responses of Gulf of Alaska plankton communities to a marine heat wave. *Deep-Sea Res. II Top. Stud. Oceanogr.* **195**, 105002 (2022).

56. U. Işkın, N. Filiz, Y. Cao, É. M. Neif, B. Öğlü, T. L. Lauridsen, T. A. Davidson, M. Søndergaard, Ü. N. Tavşanoğlu, M. Beklioğlu, Impact of nutrients, temperatures, and a heat wave on zooplankton community structure: An experimental approach. *Water* **12**, 3416 (2020).

57. H. Ullah, I. Nagelkerken, S. U. Goldenberg, D. A. Fordham, Climate change could drive marine food web collapse through altered trophic flows and cyanobacterial proliferation. *PLoS Biol.* **16**, e2003446 (2018).

58. F.-X. Fu, B. Tschitschko, D. A. Hutchins, M. E. Larsson, K. G. Baker, A. McInnes, T. Kahlke, A. Verma, S. A. Murray, M. A. Doblin, Temperature variability interacts with mean temperature to influence the predictability of microbial phenotypes. *Glob. Chang. Biol.* **28**, 5741–5754 (2022).

59. S. G. Leles, N. M. Levine, Mechanistic constraints on the trade-off between photosynthesis and respiration in response to warming. *Sci. Adv.* **9**, eadh8043 (2023).

60. M. K. Thomas, M. Aranguren-Gassis, C. T. Kremer, M. R. Gould, K. Anderson, C. A. Klausmeier, E. Litchman, Temperature–nutrient interactions exacerbate sensitivity to warming in phytoplankton. *Glob. Chang. Biol.* **23**, 3269–3280 (2017).

61. E. Bestion, C.-E. Schaum, G. Yvon-Durocher, Nutrient limitation constrains thermal tolerance in freshwater phytoplankton. *Limnol. Oceanogr. Lett.* **3**, 436–443 (2018).

62. A. Knap, A. Michaels, A. Close, D. H. A. E. Dickson, Protocols for the Joint Global Ocean Flux Study (JGOFS) Core Measurements, *JGOFS Report Nr. 19* (UNESCO, 1996).

63. I. M. Bradley, A. J. Pinto, J. S. Guest, Design and evaluation of Illumina MiSeq-compatible, 18S rRNA gene-specific primers for improved characterization of mixed phototrophic communities. *Appl. Environ. Microbiol.* **82**, 5878–5891 (2016).

64. M. Martin, Cutadapt removes adapter sequences from high-throughput sequencing reads. *EMBnet. J.* **17**, 10–12 (2011).

65. B. J. Callahan, P. J. McMurdie, M. J. Rosen, A. W. Han, A. J. A. Johnson, S. P. Holmes, DADA2: High-resolution sample inference from Illumina amplicon data. *Nat. Methods* **13**, 581–583 (2016).

66. N. Schuback, P. D. Tortell, I. Berman-Frank, D. A. Campbell, A. Ciotti, E. Courtecuisse, Z. K. Erickson, T. Fujiki, K. Halsey, A. E. Hickman, Y. Huot, M. Y. Gorbunov, D. J. Hughes, Z. S. Kolber, C. M. Moore, K. Oxborough, O. Prášil, C. M. Robinson, T. J. Ryan-Keogh, G. Silsbe, S. Simis, D. J. Suggett, S. Thomalla, D. R. Varkey, Single-turnover variable chlorophyll fluorescence as a tool for assessing phytoplankton photosynthesis and primary productivity: Opportunities, caveats and recommendations. *Front. Mar. Sci.* **8**, 690607 (2021).

67. Z. S. Kolber, O. Prasil, P. G. Falkowski, Measurements of variable chlorophyll fluorescence using fast repetition rate techniques: Defining methodology and experimental protocols. *Biochem. Biophys. Acta* **1367**, 88–106 (1998).

68. K. Oxborough, C. M. Moore, D. J. Suggett, T. Lawson, H. G. Chan, R. J. Geider, Direct estimation of functional PSII reaction center concentration and PSII electron flux on a volume basis: A new approach to the analysis of Fast Repetition Rate fluorometry (FRRf) data. *Limnol. Oceanogr. Methods* **10**, 142–154 (2012).

69. E. S. Nielsen, The use of radio-active carbon (C14) for measuring organic production in the sea. *J. du Conseil Int. pour l'Explor. de la Mer* **18**, 117–140 (1952).

70. G. B. Gloor, J. M. Macklaim, V. Pawlowsky-Glahn, J. J. Egozcue, Microbiome datasets are compositional: And this is not optional. *Front. Microbiol.* **8**, 2224 (2017).

71. P. Assmy, A. Cecilie Kvernvik, H. Hop, C. J. M. Hoppe, M. Chierici, , Seasonal plankton dynamics in Kongsfjorden during two years of contrasting environmental conditions. *Prog. Oceanogr.* **213**, 102996 (2023).
